# Supplementary material for: Female adolescents’ reproductive health decision-making capacity and contraceptive use in sub-Saharan Africa: What does the future hold?
Source: PLoS One. 2020 Jul 10;15(7):e0235601. doi: 10.1371/journal.pone.0235601 (PMC7351194; doi:10.1371/journal.pone.0235601)
Supplement: Attachment — Submitted filename: PONE-D-19-29601_comments.pdf [file pone.0235601.s002.pdf]

Overall the study shines a lens on the important challenge of ensuring adolescents have the power to make decisions about their reproductive health, including their contraceptive use. However, the study is hampered by insufficient nuance in the consideration of both its main explanatory and outcome variables. The paper would be much strengthened by a more critical discussion and reflection on this. My main critiques, described in specific comments below, refer to refining the crude measure of reproductive health decision-making capacity, considering the contraceptive need (rather than just contraceptive use) of adolescents, defining the 'sexually active' adolescent population, and expanding the outcome to modern contraception (rather than just any contraception).

Major issues

Introduction

Overall, the Introduction is very long and could be written in more clear and accessible language. The authors should consider substantially shortening the Introduction and having a clear message for each paragraph. I suggest beginning with a very brief summary on trends/regional prevalence of adolescent contraceptive use prior to the discussion of women's empowerment conceptualisation and measurement.

Methods

How was the DHS for each country selected? Was only the most recent survey used? Please provide a full list of countries and survey years (also see note in Results).

Pg 7, line 168: "same questions were posed to all women making it feasible for multi-country 169 study." This isn't quite true. While there is a standard DHS questionnaire, some adaptations are made to each survey. Were the countries and surveys selected for inclusion in this analysis based on whether they had certain questions included?

Pg 8, line 172: How was the population of sexually active adolescent girls identified in the datasets? Are these women 'in union' or was this based on time since last sexual intercourse? How was the need for contraception considered in the analysis, as some sexually active adolescents may want to become pregnant?

Pg 8, line 178: What methods are included in modern methods? Did the authors consider examining the outcome of modern method of contraception, rather than just any method of contraception? Why was modern method of contraception not used as a (secondary) outcome, particularly as using a folkloric method could reflect a desire to avoid pregnancy but an inability to access a more effective method?

For the explanatory variable of 'reproductive health decision-making capacity', how were discordant answers between the two component variables handled? Did the respondent need to have answered 'yes' to both refuse partners for sex and condom use in order to be considered to have 'reproductive health decision-making capacity'? It seems likely that women using another contraceptive method might find it strange or difficult to request that their partner, particularly in longer-term or more serious relationships, use a condom (double

protection). Did the authors conduct further analysis of how each component of the binary capacity indicator performed separately in relation to the study outcome?

The DHS also often asks questions on experience of violence and/or coercive sex. Did the authors consider other potential constructs to include in the indicator? Further discussion of the construction and limitations of this variable to capture the extremely complex concept of 'reproductive health decision-making capacity' is needed.

Pg 9, line 212-221: When combining surveys in multi-country studies to produce an overall result for sub-Saharan Africa (or any region), it is often necessary to weight country-specific estimates by the country's population. This is so that the result from a very large country like Nigeria has a greater weight in the combined effect analysis than a very small country such as Comoros. (Previous multi-country studies explaining how to apply population weights: https://doi.org/10.1016/j.jadohealth.2017.09.013 or https://doi.org/10.1111/tmi.12597) Did the authors have a reason for not applying population weights, in addition the individual survey weights standard in all DHS analysis, in the calculation of regional statistics?

Finally, I defer to a statistician, but wonder whether country is more appropriate as a random effect rather than a covariate in the models.

Results

Please provide a summary table of country, survey year, sample size of sexually active adolescents and proportion of sexually active adolescents out of all adolescents. Please also provide a summary table of the distribution of covariates. These tables can be either part of the main paper or in supplementary materials.

Figure 2: As the reproductive decision-making capacity is a simplistic measure, it would be helpful to provide estimates for each of the two components, either in the main paper or in supplementary materials.

Pg 16, lines 299-300: This links to a question in the Methods section about the need for contraception. Married adolescents may well be desiring a pregnancy so have no need for contraception.

Discussion

Overall the Discussion section is far too long and would do well to more succinctly summarise the key findings and their implications. The limitations section is insufficient in examining the severe limitations of the indicators used for both explanatory and outcome variables. 'Reproductive health decision-making capacity' is a complex construct reduced to a binary variable based on two components. The outcome of contraceptive use is likewise insufficiently nuanced to consider whether the adolescent has a need to avoid pregnancy.

Pg 21, lines 374-381: This is overly speculative and not backed up either by the results or this study or properly cited. Earlier sexual debut may itself be a marker of 'empowerment' or lack

thereof, particularly if the first sex was coerced. The DHS has further questions that could have allowed a more nuanced of this construct. Second, as the population was sexually active adolescents age 15-19, many would only very recently have had their sexual debut. Why did the authors not use more nuanced categories for age of sexual debut, particularly since all the 15-year-olds in the analysis, by definition, would fall into the <16 years at sexual debut category. What is sexual debut as the binary variable here actually telling us?

Pg 22, line 403-404: Here, and implied elsewhere in the paper, is the assertion that increasing education or empowering adolescent girls may be an effective tool for increasing adolescent contraceptive use, instead of a goal worthwhile in itself. For example: "It is imperative to strengthen existing efforts in SSA on contraceptives use among adolescent by empowering adolescents to take reproductive health decisions" (line 499-500). The authors would do well to engage more carefully in the discussion of empowerment as a means to an end (contraceptive use) rather than simply the end itself. In particular due to the paper's outcome of contraceptive use, rather than met need for contraception, the implication is that all adolescents should be using contraception, rather than all adolescents having freedom and power to choose to use contraception if they wish.

Minor issues
Suggest a careful read as typos were noted.

Introduction
Some stylistic edits needed, such as in page 4, line 94, to say 'For example, Ann Blanc [15] opined…' rather than listing the numeric reference only.

Methods
Discussion of data source and sampling could be shortened as readers can reference the individual surveys for details of the specific sampling procedures.

Pg 9, line 197: Is education level based on highest level of completed education? How were these grouped into categories? As some younger adolescents may still be in education, how did the authors consider this in the analysis?

Pg 9, line 197: Is country of origin the same as the survey country? Or does this refer to DHS questions about migration?

Pg 9, line 199: "based on their significant association with the outcome variable–contraceptives use" Was there a specific threshold used?

Pg 9, line 203: How did the authors code the occupation status of respondents still in education (students)?

Results

Figure 1: How have the countries been sorted in some way for this bar chart? It might make more sense to sort alphabetically.

Table 1: This table could be simplified by showing a total n for each row (instead of a sample size for ‘no’ and for ‘yes’). Only the percentage of ‘yes’ is needed (as the ‘no’ for any contraceptive use is just the corresponding fraction). Please provide confidence intervals.

Pg 16, line 290: Is there a reason the comparison or reference country is Angola?

Discussion
Pg 19, line 323: Could the very different estimate be related to the crude measure of reproductive decision-making capacity measure used in this study?

Pg 23, line 430: “Female adolescents’ ability to make reproductive health decisions was associated with contraceptive use, which in turn could be connected with perceived unmet need.” This point about contraceptive use connected with perceived unmet need is confusing.
