# Supplementary material for: Female adolescents’ reproductive health decision-making capacity and contraceptive use in sub-Saharan Africa: What does the future hold?
Source: PLoS One. 2020 Jul 10;15(7):e0235601. doi: 10.1371/journal.pone.0235601 (PMC7351194; doi:10.1371/journal.pone.0235601)
Supplement: Attachment — Submitted filename: Review Comments_Plos One.docx [file pone.0235601.s001.docx]

I acknowledge the authors for a comprehensive and in terms of language, a well-written paper. The paper was easy to follow. Common, yet important limitations of studies of such types involving sexual and reproductive health (SRH) were detailed in the “Limitation” section. Few comments I have are as follows:

1. Important things to mention in the last part of the “Introduction” section always are to briefly mention similar studies, what they lacked (the research gaps) and which gaps the current study aims to fulfill. This would make clear for the readers how the study is different from other studies with similar research objective. This important aspect is seen to be missing in the “Introduction” part.

An example for it would be: In the material and method section, the authors mention to have followed previous studies (referenced 33 and 34) (Line no. 190). We see the topic of these two studies are similar to the current study, and study of such types are existent, however, the authors neither mention this study in the “Introduction” nor the gaps these studies had and the gaps that the current study attempts to cover. Another similar comment when the authors directly mention other two studies (reference 42, 43) (L.352) in the discussion and compare their results with them.

2. Although the study covered many aspects determining the reproductive decision making and contraceptive use, some interesting analysis could have been carried out additionally. I suggest a subgroup analysis grouping the countries with SRH or contraceptive intervention and the countries without. I believe that reproductive decision alone cannot facilitate contraceptive use (To explain, even if one has the reproductive decision making capacity but no availability of contraceptive, he/she would not be able to use contraceptives despite the will. On the other hand, if a person has very easy access to contraceptives, a slight decision making capacity would also be enough for contraceptive use).
3. L: 258-270: I suggest mentioning the CI of the given results. (CI across different countries).

Minor comments:

1. I would suggest replacing the word “downward”(L. 119)
2. L.127-130, I would consider rewriting the sentence with minor changes to make it clearer (e.g. parental communication is not related to high risk of adolescent pregnancy but I believe poor parental communication is)
3. L. 102: reference missing for –“ despite sizable body of literature”
4. L.106, 358 etc. I would consider putting the reference at the end of the sentence or after some words or data from the study but not directly after one word – “According” or “Similarly”. Eg: “According to a study, these persons ……. [18] “OR “According to a study [18], these persons ……. “
5. L. 332,334: I suggest restructuring the last part of the sentence
