# Supplementary material for: Female adolescents’ reproductive health decision-making capacity and contraceptive use in sub-Saharan Africa: What does the future hold?
Source: PLoS One. 2020 Jul 10;15(7):e0235601. doi: 10.1371/journal.pone.0235601 (PMC7351194; doi:10.1371/journal.pone.0235601)
Supplement: Attachment — Submitted filename: edited PONE-D-19-29601_R1.docx [file pone.0235601.s004.docx]

PLOS ONE

Female adolescents’ reproductive health decision-making capacity and contraceptive use in Sub-Saharan Africa: What does the future hold?

--Manuscript Draft--

| Manuscript Number: | PONE-D-19-29601R1 |
| --- | --- |
| Article Type: | Research Article |
| Full Title: | Female adolescents’ reproductive health decision-making capacity and contraceptive use in Sub-Saharan Africa: What does the future hold? |
| Short Title: | Female adolescents’ reproductive health decision-making capacity and contraceptive use in sub-Saharan Africa |
| Corresponding Author: | Abdul-Aziz Seidu, Mphil  University of Cape Coast  Cape Coast, Central Region GHANA |
| Keywords: | Adolescents; Decision-making capacity; contraceptives; Sub-Saharan Africa |
| Abstract: | Introduction  Given the social, economic and health imports of early parenthood, unintended pregnancy, and the risks of HIV infection and subsequent transmission, there is an urgent need to understand how adolescents make sexual and reproductive decisions regarding contraceptive use. This study sought to assess the influence of female adolescents’ reproductive health decision-making capacity on their contraceptive usage.  Materials and methods  Data was obtained from pooled current Demographic and Health Surveys [DHS] conducted in 32 countries in Sub-Saharan Africa [SSA]. The unit of analysis for this study was sexually active female adolescents [n= 11,474 ]. Bivariate and multivariate analysis were conducted using Pearson chi-square tests and binary logistic regression respectively. All analysis were performed using STATA version 14.2. Results were presented using Odds Ratios [OR] and adjusted Odds Ratios [AOR].  Results  The results showed that 71.9% of the adolescents had the capacity to make reproductive health decisions. It was also found that overall prevalence of contraceptive use was 20.07% and it ranged from 2.3% in Chad to 48.8% in Rwanda. In the multivariate analysis, adolescents who had the capacity to take reproductive health decisions had higher odds to use contraceptives [AOR = 1.41; CI = 1.24–1.60, p < 0.001]. Compared to Chad, female adolescents in Zimbabwe had the highest odds of using contraceptives [AOR = 36.50 ; CI = 8.98 – 148.3 , p < 0.001]. The odds of contraceptive use among female adolescents increased with age, with those aged 19 years having the highest likelihood of using contraceptives [AOR = 3.59 ; CI = 2.56 – 5.04 , p < 0.001]. The results further showed that the higher the level of education, the more likely some female adolescents will use contraceptives and this was more predominant among individuals with secondary education [AOR = 2.46 ; CI = 2 .04 – 2.98 , p < 0.001]. Conclusion  The use of general and modern contraceptives among adolescents in SSA remains low . Therefore, there is a need to strengthen existing efforts on  contraceptives usage among adolescents in SSA. This can be achieved by empowering young females particularly in the rural areas where level of literacy is very low to take positive reproductive health decisions to prevent teenage pregnancy, HIV and other sexually transmitted infections, reduce maternal mortality and early childbirth. |
| Order of Authors: | Bright Opoku Ahinkorah |
|  | John Elvis Hagan |
|  | Abdul-Aziz Seidu, Mphil |
|  | Francis Sambah |
|  |  |

|  | Faustina Adoboi |
| --- | --- |
|  | Thomas Schack |
|  | Eugene Budu |
| Response to Reviewers: | AUTHOR’S RESPONSE TO REVIEWS  Title: Female adolescents’ reproductive health decision-making capacity and contraceptive use in sub-Saharan Africa: What does the future hold?  Dear Editor and Reviewers,  Thank you for your email dated 9th March 2020 enclosing the reviewer’s comments. On behalf of all authors, I convey our gratitude to you for the critical and constructive review that has led to the massive improvement of our paper entitled “Female adolescents’ reproductive health decision-making capacity and contraceptive use in sub-Saharan Africa: What does the future hold?”. We have carefully reviewed the comments from all the three reviewers and have revised the manuscript accordingly. Our responses are given in a point-by-point manner below.Most of the changes have been indicated in yellow colour. We believe the manuscript has improved substantively and will be published in your reputable journal.  Version 1: PONE-D-19-29601  Date:4/4/2020  REVIEWER #1:  Comment: In the methodology, the author/authors suggested the use of Bivariate and multivariable analysis which include Pearson chi-square tests and binary logistic regression respectively, however, binary logistic regression model in most cases is misleading in interpreting the complex relationships of this nature. The authors need to demonstrate how they deal with existence of multicollinearity problems which obviously made the model deficiency.  I feel that the problem of multicollinearity cannot be undermined in this study because it can lead the authors into making incorrect conclusion about relationships between responsive and explanatory variables. Therefore, I would suggest that the authors should consider adopting Principle component analysis (PCA) in addition to regression approaches to avoid this problem. The PCA is a multivariate technique which help to understand the underlying data structure and to form a smaller number of uncorrelated new variables. In other words, PCA reduces number of predictors to avoid multicollinearity problem and it is highly recommended due to its ability to identify a small number of derived variables from a larger number of original variables in order to simplify the subsequent analysis of the data.  Response: Before results were interpreted, multicollinearity between explanatory variables was assessed through the variance inflation factors (VIF) at a reference value of 5 (We have attached this as a supplementary file. (page 7)  REVIEWER #2:  I acknowledge the authors for a comprehensive and in terms of language, a wellwritten paper. The paper was easy to follow. Common, yet important limitations of studies of such types involving sexual and reproductive health (SRH) were detailed in the “Limitation” section. Few comments I have are as follows:  Comment 1. Important things to mention in the last part of the “Introduction” section always are to briefly mention similar studies, what they lacked (the research gaps) and which gaps the current study aims to fulfil. This would make clear for the readers how the study is different from other studies with similar research objective. This important aspect is seen to be missing in the “Introduction” part. An example for it would be: In the material and method section, the authors mention to have followed previous studies (referenced 33 and 34) (Line no. 190). We see the topic of these two studies are similar to the current study, and study of such types are existent, however, the authors neither mention this study in the “Introduction” nor the gaps these studies had and the gaps that the current study attempts to cover. Another similar comment when the authors directly mention other two studies (reference 42, 43) (L.352) in the |

|  | discussion and compare their results with them.  Response: We have revised the introduction of our manuscript to take into consideration these issues (see Page 3-5).  Comment 2. Although the study covered many aspects determining the reproductive decision making and contraceptive use, some interesting analysis could have been carried out additionally. I suggest a subgroup analysis grouping the countries with SRH or contraceptive intervention and the countries without. I believe that reproductive decision alone cannot facilitate contraceptive use (To explain, even if one has the reproductive decision making capacity but no availability of contraceptive, he/she would not be able to use contraceptives despite the will. On the other hand, if a person has very easy access to contraceptives, a slight decision making capacity would also be enough for contraceptive use).  Response: Even though sexual and reproductive health interventions are not evenly distributed in sub-Saharan Africa, each country has instituted various policies to tackle contraceptive usage: Please see (Liang, M., Simelane, S., Fillo, G. F., Chalasani, S., Weny, K., Canelos, P. S., ... & Michielsen, K. (2019). The state of adolescent sexual and reproductive health. Journal of Adolescent Health, 65(6), S3-S15.  Melesse, D. Y., Mutua, M. K., Choudhury, A., Wado, Y. D., Faye, C. M., Neal, S., & Boerma, T. (2020). Adolescent sexual and reproductive health in sub-Saharan Africa: who is left behind?. BMJ Global Health, 5(1).; https://www.afro.who.int/healthtopics/sexual-and-reproductive-health)  Comment: 3. L: 258-270: I suggest mentioning the CI of the given results. (CI across different countries).  Response: We have presented the results as forest plots and the results are now showing their respective 95% Cis (Please see Fig 1-4).  Minor comments:  Comment: 1. I would suggest replacing the word “downward”(L. 119)  2. L.127-130, I would consider rewriting the sentence with minor changes to make it clearer (e.g. parental communication is not related to high risk of adolescent pregnancy but I believe poor parental communication is)  Response: We have revised the background based on the recommendations made by Reviewer 3 and all these issues have been addressed (see Page 3-5).  Comment: 3. L. 102: reference missing for –“ despite sizable body of literature”  Response: We have revised the background based on the recommendations made by Reviewer 3 (see Page 3-5).  Comment: 4. L.106, 358 etc. I would consider putting the reference at the end of the sentence or after some words or data from the study but not directly after one word –  “According” or “Similarly”. Eg: “According to a study, these persons ……. [18] “OR  “According to a study [18], these persons ……. “  5. L. 332,334: I suggest restructuring the last part of the sentence.  Response: We have taken note of this and revised some of these statements in the manuscript.  REVIEWER #3:  The statistical analysis is fairly simple compared to the conceptual challenges presented in the study. There is one issue that may require the opinion of a statistician regarding the inclusion of country as a random effect in the model. Overall the study shines a lens on the important challenge of ensuring adolescents have the power to make decisions about their reproductive health, including their contraceptive use. |
| --- | --- |

|  | However, the study is hampered by insufficient nuance in the consideration of both its main explanatory and outcome variables. The paper would be much strengthened by a more critical discussion and reflection on this. My main critiques, described in specific comments below, refer to refining the crude measure of reproductive health decisionmaking capacity, considering the contraceptive need (rather than just contraceptive use) of adolescents, defining the ‘sexually active’ adolescent population, and expanding the outcome to modern contraception (rather than just any contraception).  Major issues Introduction  Comment: Overall, the Introduction is very long and could be written in more clear and accessible language. The authors should consider substantially shortening the  Introduction and having a clear message for each paragraph. I suggest beginning with a very brief summary on trends/regional prevalence of adolescent contraceptive use prior to the discussion of women’s empowerment conceptualisation and measurement.  Response: We have revised the background taking on board all the suggestions (see Page 3-5).  Methods  Comment: How was the DHS for each country selected? Was only the most recent survey used? Please provide a full list of countries and survey years (also see note in Results).  Response: We mentioned in the methods how each DHS was selected and have provided a Table that contains the countries and their survey years and the sample (See Table 1).  Comment: Pg 7, line 168: “same questions were posed to all women making it feasible for multi-country 169 study.” This isn’t quite true. While there is a standard DHS questionnaire, some adaptations are made to each survey. Were the countries and surveys selected for inclusion in this analysis based on whether they had certain questions included?  Response: The countries were selected because they had similar variables. “The DHS contain both ‘core’ and optional questionnaires (modules). Core questionnaires cover basic demographic and health content, including marriage, fertility, family planning, reproductive health and child health, whereas additional modules contain special topics, including maternal mortality, men’s survey, anthropometry (height and weight measurement), anaemia blood testing, gender/domestic violence, malaria, maternal mortality, tobacco use, chronic illnesses and other biomarkers” Consequently, each survey is tailored to the needs of a particular country while containing several basic components that are comparable across all countries ( Corsi, Neuman, Finlay & Subramanian, 2012 p.1606). Corsi, D. J., Neuman, M., Finlay, J. E., & Subramanian, S. V. (2012). Demographic and health surveys: a profile. International journal of epidemiology, 41(6), 1602-1613.  Comment: Pg 8, line 172: How was the population of sexually active adolescent girls identified in the datasets? Are these women ‘in union’ or was this based on time since last sexual intercourse? How was the need for contraception considered in the analysis, as some sexually active adolescents may want to become pregnant? Response: The population for sexually active adolescents were determined by those who indicated they have ever had sex and mentioned their age at first sex as used in previous studies (Appiah et al 2020). Please we acknowledge the need for contraception as a limitation in our study. (see page 26)  Comment: Pg 8, line 178: What methods are included in modern methods? Did the authors consider examining the outcome of modern method of contraception, rather than just any method of contraception? Why was modern method of contraception not used as a (secondary) outcome, particularly as using a folkloric method could reflect a desire to avoid pregnancy but an inability to access a more effective method?  Response: The modern methods included female sterilization, male sterilization, |
| --- | --- |

|  | intrauterine contraceptive device (IUD), contraceptive injection, contraceptive implants (Norplant), contraceptive pill, condoms, emergency contraception, standard day method (SDM), vaginal methods (foam, jelly, suppository), lactational amenorrhea method (LAM), country-specific modern methods and respondent-mentioned other modern contraceptive methods (including cervical cap, contraceptive sponge, and others). Aside examining contraceptive use in general, we also examined the factors associated with modern contraceptive use (secondary outcome variable) (see Table 3  ).  Comment: For the explanatory variable of ‘reproductive health decision-making capacity’, how were discordant answers between the two component variables handled? Did the respondent need to have answered ‘yes’ to both refuse partners for sex and condom use in order to be considered to have ‘reproductive health decisionmaking capacity’? It seems likely that women using another contraceptive method might find it strange or difficult to request that their partner, particularly in longer-term or more serious relationships, use a condom (double protection). Did the authors conduct further analysis of how each component of the binary capacity indicator performed separately in relation to the study outcome? The DHS also often asks questions on experience of violence and/or coercive sex. Did the authors consider other potential constructs to include in the indicator? Further discussion of the construction and limitations of this variable to capture the extremely complex concept of ‘reproductive health decision-making capacity’ is needed.  Response: We followed the measurement of ‘reproductive health decision-making capacity’ by previous studies:  Darteh EK, Doku DT, Esia-Donkoh K. Reproductive health decision-making among Ghanaian women. Reproductive health. 2014;11(1):23  Darteh, E. K. M., Dickson, K. S., & Doku, D. T. (2019). Women’s reproductive health decision-making: A multi-country analysis of demographic and health surveys in subSaharan Africa. PloS one, 14(1)  Seidu AA, Ahinkorah BO, Agbemavi W, Amu H, Bonsu F. Reproductive health decision-making capacity and pregnancy termination among Ghanaian women:  Analysis of the 2014 Ghana demographic and health survey. Journal of Public Health. 2019:1-0.  We have acknowledged limitation in the construction of ‘reproductive health decisionmaking capacity’ in our manuscript and recommend further studies to look at these limitations to refine its measurement (page 26.)  Comment: Pg 9, line 212-221: When combining surveys in multi-country studies to produce an overall result for sub-Saharan Africa (or any region), it is often necessary to weight country-specific estimates by the country’s population. This is so that the result from a very large country like Nigeria has a greater weight in the combined effect analysis than a very small country such as Comoros. (Previous multi-country studies explaining how to apply population  weights:https://doi.org/10.1016/j.jadohealth.2017.09.013 or  https://doi.org/10.1111/tmi.12597) Did the authors have a reason for not applying population weights, in addition the individual survey weights standard in all DHS analysis, in the calculation of regional statistics? Finally, I defer to a statistician, but wonder whether country is more appropriate as a random effect rather than a covariate in the models.  Response: We applied both population weight and sample weights (see page 10) .Again a test of heterogeneity of the DHS data obtained for the different countries showed a high level of inconsistency (I2 > 50%) thereby warranting the use of a random effect model in all the meta-analysis (see Figures 1, 2, 3 and 4), but at the regression models as used in previous studies, country was considered a covariate:  Darteh, E. K. M., Dickson, K. S., & Doku, D. T. (2019). Women’s reproductive health decision-making: A multi-country analysis of demographic and health surveys in sub-  Saharan Africa. PloS one, 14(1); |
| --- | --- |

|  | Ameyaw, E. K., Budu, E., Sambah, F., Baatiema, L., Appiah, F., Seidu, A. A., & Ahinkorah, B. O. (2019). Prevalence and determinants of unintended pregnancy in sub-Saharan Africa: A multi-country analysis of demographic and health surveys. PloS one, 14(8).  Results  Comment: Please provide a summary table of country, survey year, sample size of sexually active adolescents and proportion of sexually active adolescents out of all adolescents. Please also provide a summary table of the distribution of covariates. These tables can be either part of the main paper or in supplementary materials. Response: This has been provided. See Table 1.  Comment: Figure 2: As the reproductive decision-making capacity is a simplistic measure, it would be helpful to provide estimates for each of the two components, either in the main paper or in supplementary materials.  Response: We have provided the estimates in the forest plots. See Figure 2-4  Comment: Pg 16, lines 299-300: This links to a question in the Methods section about the need for contraception. Married adolescents may well be desiring a pregnancy so have no need for contraception.  Response: We have acknowledged this as a limitation in the paper (page 26.)  Discussion  Comment: Overall the Discussion section is far too long and would do well to more succinctly summarise the key findings and their implications. The limitations section is insufficient in examining the severe limitations of the indicators used for both explanatory and outcome variables. ‘Reproductive health decision-making capacity’ is a complex construct reduced to a binary variable based on two components. The outcome of contraceptive use is likewise insufficiently nuanced to consider whether the adolescent has a need to avoid pregnancy.  Response: We have revised the discussion and acknowledged the limitations in measurement of both the explanatory and outcome variables( See page 21-27).  Comment: Pg 21, lines 374-381: This is overly speculative and not backed up either by the results or this study or properly cited. Earlier sexual debut may itself be a marker of ‘empowerment’ or lack thereof, particularly if the first sex was coerced. The DHS has further questions that could have allowed a more nuanced of this construct. Second, as the population was sexually active adolescents age 15-19, many would only very recently have had their sexual debut. Why did the authors not use more nuanced categories for age of sexual debut, particularly since all the 15- year-olds in the analysis, by definition, would fall into the <16 years at sexual debut category. What is sexual debut as the binary variable here actually telling us?  Response: We have revised the discussion.  Comment: Pg 22, line 403-404: Here, and implied elsewhere in the paper, is the assertion that increasing education or empowering adolescent girls may be an effective tool for increasing adolescent contraceptive use, instead of a goal worthwhile in itself. For example: “It is imperative to strengthen existing efforts in SSA on contraceptives use among adolescent by empowering adolescents to take reproductive health decisions” (line 499-500). The authors would do well to engage more carefully in the discussion of empowerment as a means to an end (contraceptive use) rather than simply the end itself. In particular due to the paper’s outcome of contraceptive use, rather than met need for contraception, the implication is that all adolescents should be using contraception, rather than all adolescents having freedom and power to choose to use contraception if they wish.  Response: We have revised this section. See page 24) |
| --- | --- |

|  | Minor issues  Comment: Suggest a careful read as typos were noted.  Response: We have reviewed and edited the manuscript extensively. The manuscript has also been reviewed by a native English speaker.  Introduction  Comment: Some stylistic edits needed, such as in page 4, line 94, to say ‘For example, Ann Blanc [15] opined…’ rather than listing the numeric reference only. Response: These issues have been rectified.  Methods  Comment: Discussion of data source and sampling could be shortened as readers can reference the individual surveys for details of the specific sampling procedures. Response: This section has been shortened.  Comment: Pg 9, line 197: Is education level based on highest level of completed education? How were these grouped into categories? As some younger adolescents may still be in education, how did the authors consider this in the analysis? Response: Thank you. This is on highest level of education completed  Comment: Pg 9, line 197: Is country of origin the same as the survey country? Or does this refer to DHS questions about migration?  Response: This is referring to surveyed country. This has been clarified in the methods section of the manuscript.  Comment: Pg 9, line 199: “based on their significant association with the outcome variable–contraceptives use” Was there a specific threshold used?  Response: The threshold was that all those studies used nationally-representative survey data.  Comment: Pg 9, line 203: How did the authors code the occupation status of respondents still in education (students)?  Response: Education was captured based on highest level completed.  Results  Comment: Figure 1: How have the countries been sorted in some way for this bar chart? It might make more sense to sort alphabetically.  Response: This has been presented with forest plot (see Figure 1-4).  Comment: Table 1: This table could be simplified by showing a total n for each row  (instead of a sample size for ‘no’ and for ‘yes’). Only the percentage of ‘yes’ is needed (as the ‘no’ for any contraceptive use is just the corresponding fraction). Please provide confidence intervals.  Response: This has been presented with forest plot (see Figure 1-4).  Comment: Pg 16, line 290: Is there a reason the comparison or reference country is Angola?  Response: We have revised this. We have used the country with the lowest proportion (Zambia) as the reference category.  Discussion  Comment: Pg 19, line 323: Could the very different estimate be related to the crude measure of reproductive decision-making capacity measure used in this study? Response: This section has been revised.  Comment: Pg 23, line 430: “Female adolescents’ ability to make reproductive health decisions was associated with contraceptive use, which in turn could be connected with perceived unmet need.” This point about contraceptive use connected with perceived unmet need is confusing.  Response: This statement has been revised. |
| --- | --- |
| Additional Information: |  |

| Question | Response |
| --- | --- |
| Financial Disclosure  Enter a financial disclosure statement that describes the sources of funding for the work included in this submission. Review the [submission guidelines](http://journals.plos.org/plosone/s/submission-guidelines#loc-financial-disclosure-statement) for detailed requirements. View published research articles from [PLOS ONE](http://journals.plos.org/plosone/) for specific examples.  This statement is required for submission and will appear in the published article if the submission is accepted. Please make sure it is accurate. | The author(s) received no specific funding for this work. |
| Unfunded studies  Enter: The author(s) received no specific funding for this work.  Funded studies  Enter a statement with the following details:   - Initials of the authors who received each award - Grant numbers awarded to each author - The full name of each funder - URL of each funder website - Did the sponsors or funders play any role in the study design, data collection and analysis, decision to publish, or preparation of the manuscript? - NO - Include this sentence at the end of your statement: The funders had no role in   study design, data collection and analysis, decision to publish, or preparation of the manuscript.   - YES - Specify the role(s) played. |  |
| * typeset |  |
| Competing Interests  Use the instructions below to enter a competing interest statement for this submission. On behalf of all authors, disclose any [competing interests](http://journals.plos.org/plosone/s/competing-interests) that could be perceived to bias this work—acknowledging all financial support and any other relevant financial or nonfinancial competing interests.  This statement will appear in the published article if the submission is | The authors have declared that no competing interests exist. |

| accepted. Please make sure it is accurate. View published research articles from [PLOS ONE](http://journals.plos.org/plosone/) for specific examples. |  |
| --- | --- |
| NO authors have competing interests  Enter: The authors have declared that no competing interests exist.  Authors with competing interests  Enter competing interest details beginning with this statement:  I have read the journal's policy and the authors of this manuscript have the following competing interests: [insert competing interests here] |  |
| * typeset |  |
| Ethics Statement  Enter an ethics statement for this submission. This statement is required if the study involved:   - Human participants - Human specimens or tissue - Vertebrate animals or cephalopods - Vertebrate embryos or tissues - Field research   Write "N/A" if the submission does not require an ethics statement.  General guidance is provided below. Consult the [submission guidelines](http://journals.plos.org/plosone/s/submission-guidelines#loc-guidelines-for-specific-study-types) for detailed instructions. Make sure that all information entered here is included in the Methods section of the manuscript. | The DHS surveys obtain ethical clearance from the Ethics Committee of ORC Macro  Inc. as well as Ethics Boards of partner organisations of the various countries such the Ministries of Health. During each of the surveys, either written or verbal consent was provided by the women. Since the data was not collected by the authors of this manuscript, official permission was sought from MEASURE DHS website and access to the data was provided after our intent for the request was assessed and approved on 3rd April, 2019. Data is available on https://dhsprogram.com/data/availabledatasets.cfm |

| Format for specific study types  Human Subject Research (involving human participants and/or tissue)   - Give the name of the institutional review board or ethics committee that approved the study - Include the approval number and/or a statement indicating approval of this research - Indicate the form of consent obtained (written/oral) or the reason that consent was not obtained (e.g. the data were analyzed anonymously)   Animal Research (involving vertebrate animals, embryos or tissues)   - Provide the name of the Institutional Animal Care and Use Committee (IACUC) or other relevant ethics board that reviewed the study protocol, and indicate whether they approved this research or granted a formal waiver of ethical approval - Include an approval number if one was obtained - If the study involved non-human primates, add additional details about animal welfare and steps taken to ameliorate suffering - If anesthesia, euthanasia, or any kind of animal sacrifice is part of the study, include briefly which substances and/or methods were applied   Field Research  Include the following details if this study involves the collection of plant, animal, or other materials from a natural setting:   - Field permit number - Name of the institution or relevant body that granted permission |  |
| --- | --- |
|  |  |
| Data Availability  Authors are required to make all data underlying the findings described fully available, without restriction, and from the time of publication. PLOS allows rare exceptions to address legal and ethical concerns. See the [PLOS Data Policy](http://journals.plos.org/plosone/s/data-availability) and [FAQ](http://journals.plos.org/plosone/s/data-availability#loc-faqs-for-data-policy) for detailed information. | Yes - all data are fully available without restriction |

| A Data Availability Statement describing where the data can be found is required at submission. Your answers to this question constitute the Data Availability Statement and will be published in the article, if accepted. |  |
| --- | --- |
| Important: Stating ‘data available on request from the author’ is not sufficient. If your data are only available upon request, select ‘No’ for the first question and explain your exceptional situation in the text box. |  |
| Do the authors confirm that all data underlying the findings described in their manuscript are fully available without restriction? |  |
| Describe where the data may be found in full sentences. If you are copying our sample text, replace any instances of XXX with the appropriate details.   - If the data are held or will be held in a public repository, include URLs, accession numbers or DOIs. If this information will only be available after acceptance, indicate this by ticking the box below. For example: All XXX files   are available from the XXX database (accession number(s) XXX, XXX.).   - If the data are all contained within the manuscript and/or Supporting Information files, enter the following: All relevant data are within the manuscript and its Supporting Information files. - If neither of these applies but you are able to provide details of access elsewhere, with or without limitations, please do so. For example:   Data cannot be shared publicly because of [XXX]. Data are available from the XXX Institutional Data Access / Ethics Committee (contact via XXX) for researchers who meet the criteria for access to confidential data.  The data underlying the results presented in the study are available from (include the name of the third party | Data is available on https://dhsprogram.com/data/available-datasets.cfm. |

| and contact information or URL).  • This text is appropriate if the data are owned by a third party and authors do not have permission to share the data.  * typeset |  |
| --- | --- |
| Additional data availability information: |  |

Manuscript	[Click here to access/download;Manuscript;Clean file_RHDM_CU_6.4.2020.docx](https://www.editorialmanager.com/pone/download.aspx?id=26363684&guid=d04275c6-50a7-4252-8b4f-d45715f6c1d0&scheme=1)

1	**Female adolescents’ reproductive health decision-making capacity**

# 2 and contraceptive use in sub-Saharan Africa: What does the future

3	**hold?**

4

5 Bright Opoku Ahinkorah^1^, John Elvis Hagan Jr.^2,4^, Abdul-Aziz Seidu^3^*, Francis Sambah^2^, 6 Faustina Adoboi^5^, Thomas Schack^4^, Eugene Budu^3^

7

8 ^1^The Australian Centre for Public and Population Health Research [ACPPHR], Faculty of
9 Health, University of Technology Sydney, Australia
10^2^Department of Health, Physical Education, and Recreation, University of Cape Coast, Cape 11	Coast, Ghana
12 ^3^Department of Population and Health, University of Cape Coast, Cape Coast, Ghana
13 ^4^Neurocognition and Action-Biomechanics-Research Group, Faculty of Psychology and
14 Sport Sciences, Bielefeld University, Bielefeld-Germany
15 ^5^Cape Coast Nursing and Midwifery Training College, Cape Coast, Ghana

16

## 17 *Corresponding author

18 Abdul-Aziz Seidu
19 Email: abdul-aziz.seidu@stu.ucc.edu.gh

20

21

22

23

24

# Abstract

## Introduction

Given the social, economic and health imports of early parenthood, unintended pregnancy, and the risks of HIV infection and subsequent transmission, there is an urgent need to understand how adolescents make sexual and reproductive decisions regarding contraceptive use. This study sought to assess the influence of female adolescents’ reproductive health decisionmaking capacity on their contraceptive usage.

## Materials and methods

Data was obtained from pooled current Demographic and Health Surveys [DHS] conducted in 32 countries in sub-Saharan Africa [SSA]. The unit of analysis for this study was sexually active female adolescents [n=11,474]. Bivariate and multivariable analyses were conducted using Pearson chi-square tests and binary logistic regression respectively. All analyses were performed using STATA version 14.2. Results were presented using Odds Ratios [OR] and adjusted Odds Ratios [AOR]. **Results**

The results showed that 71.9% of the adolescents had the capacity to make reproductive health decisions. It was also found that overall prevalence of contraceptive use was 20.07% and it ranged from 2.3% in Chad to 48.8% in Rwanda. In the multivariable analysis, adolescents who had the capacity to take reproductive health decisions had higher odds to use contraceptives [AOR = 1.41; CI = 1.24–1.60, p < 0.001]. Compared to Chad, female adolescents in Zimbabwe had the highest odds of using contraceptives [AOR = 36.50; CI = 8.98–148.3, p < 0.001]. The odds of contraceptive use among female adolescents increased with age, with those aged 19 years having the highest likelihood of using contraceptives [AOR = 3.59; CI = 2.56–5.04, p <

0.001]. The results further showed that the higher the level of education, the more likely some female adolescents will use contraceptives and this was more predominant among individuals with secondary education [AOR = 2.46; CI = 2.04–2.98, p < 0.001].

## Conclusion

Utilisation of contraceptives in general and modern contraceptives in particular remains low in among adolescents in SSA. Strengthening existing efforts in SSA on contraceptives usage among adolescents by empowering these young females to take positive reproductive health decisions is imperative. This enlightenment should target younger female adolescents, specifically, those in rural areas, and without formal education as well as those who initiate sexual intercourse between age 16 and 19 years. These can help reduce teenage pregnancy, prevent some infections, reduce maternal mortality and early childbirth and also help achieve SDG three.

**Keywords: Adolescents; Decision-making capacity; Contraceptives; Sub-Saharan**

**Africa**

# Introduction

Empirical data from Sub-Sahara Africa (SSA) suggest that a high proportion of 15- to 19-yearolds are sexually active and at risk of contracting HIV, other STIs or of unplanned pregnancy because of lack of contraceptive use [1,2]. Although, recent statistics show that contraceptive use among young adult women in SSA has improved in line with global trends [3,4], young women in these age brackets disproportionately use contraceptives, especially short-term methods (e.g., condoms [4]). Given the heterogeneous nature of the SSA region, sub-regional variations exist in young adult women’s contraceptive use. Generally, among women of reproductive age (i.e., 15–49), reported modern contraceptive use between 2005 and 2015 is highest (54.3%) in Southern Africa (i.e., Botswana, Lesotho, Namibia, South Africa and

Swaziland), followed by Northern Africa (i.e., Algeria, Egypt, Libya, Morocco, Sudan, and Tunisia). The moderate to lowest trends are recorded in Eastern Africa (27.2%), Western (15%), and Central Africa (12%) countries [5]. According to Bundy et al. [6], it is possible that these developments could be similar for young adult populations across the region. This assertion explains why on the average, 26–30% of presently married women age 15–24 and 40–41% of recently unmarried women age 15–24 report an unmet need for family planning in the West/Central and East/South regions of Africa [7].

Scholarly information reveals that empowering women is generally seen to be critical for effective implementation of family planning and reduction of HIV transmission [8,9,10]. Empowered women are often associated with the desire for fewer children, readily access more health services, avoid risky sexual partners, effectively strategize their births [10], and utilize condoms as HIV and STI prevention method [11,12]. Comparatively, enhanced health and contraceptive accessibility and usage may empower women [13]. For instance, women who accessed and utilized diaphragms applauded their recent aptitude to demonstrate safer sexual behaviour in Kenya [14]. Similarly, Blanc [15] showed that the steadiness of power in sexual relations has association with usage of health services and reproductive health outcomes. Despite a sizeable body of literature demonstrating the association between various dimensions of women empowerment on contraceptive usage, only few studies have explicitly measured the association between women health decision making capacity and contraceptive use in SSA [16,17,18,19]. Bankole and Singh (16) found that contraceptive usage was more pronounced when both couples decided to halt childbearing and reported lowest when both partners desired more children. Do and Kurimoto (17) later showed positive association between women empowerment score and contraceptive use in selected countries (i.e., Ghana, Namibia, Uganda, Zambia and Zimbabwe).

Despite these studies contributing to existing literature on women’s reproductive health decision making and additional knowledge to the limited information on sexual and reproductive health issues in SSA, some flaws should not be overlooked. Specifically, the National Demographic Survey data used by some of the researchers (e.g., Bankole and Singh; Do and Kurimoto) were compiled from different years and somewhat old, therefore may contain inconsistent variables with measurement errors and trends for effective analytical comparisons. Hence, findings from these studies may have been either over-exaggerated or under reported by the authors. Similarly, findings noted by Darteh et al., [18] and Seidu et al., [19] were very limited in scope (i.e., data from one country) and cannot be used for multi-country comparisons and make generalizations across SSA. Even more compelling for additional research is that, in many SSA countries, women’s right to health, including sexuality has been infringed because of socio-cultural barriers (e.g., most decisions taken by men, [20,21,22]).

Given the sparse literature on reproductive health decision making among adolescents in SSA, it is for strategic programmes and policy standpoint to better understand female adolescents’ health decision making capacity and contraceptive use behaviour for their later healthy sexual and reproductive lives [23,24]. There is general call for more research, policy, and programming attention that meet the reproductive health needs of young adult African women [25,26,4]. Therefore, the aim of this multi-country study was to assess the influence of female adolescents’ reproductive health decision-making capacity on their contraceptive usage.

We anticipate that female adolescents’ reproductive health decision-making capacity would shape their socio-cultural norms (e.g., subjective gender role orientations) and positively be related to contraceptive use.

# Materials and methods

## Data source

The study made use of pooled data from current Demographic and Health Survey (DHS) conducted from January 1, 2010 and December 31, 2018 in 32 countries in sub- Saharan Africa.

Table 1 provides a full list of the countries included in the survey and their survey years. The 32 countries were used in the study because they had complete data on the variables of interest for this study. The study included the countries under the DHS program in order to provide a holistic and in-depth evidence of the relationship between reproductive health decision making capacity and contraceptive use in SSA. DHS is a nationwide survey collected every five-year period across low and middle-income countries. The survey is representative of each of these countries. Women’s files were used for our study and these files possess the responses by women aged 15–49. The survey targets core maternal and child health indicators such as unintended pregnancy, contraceptive use, skilled birth attendance, immunization among under-fives, and intimate partner violence. Stratified dual-stage sampling approach was employed and the same questions were posed to all women making it feasible for multi-country study. Selection of clusters (i.e., enumeration areas [EAs]) is the first step in the sampling process followed by systematic household sampling within the selected EAs. For the purpose of this study, only sexually active adolescent girls (15-19 years) who had complete cases on all the variables of interest were used for the study (N = 11,474). Detailed description of the study sample is shown in Table 1.

### Table 1: Description of the study sample

| **Country** | **Survey Year** | **Sample (N)** | **Sample (%)** |
| --- | --- | --- | --- |
| Angola | 2015-16 | 660 | 19.6 |
| Burkina Faso | 2010 | 299 | 8.9 |
| Benin | 2017-18 | 273 | 8.2 |
| Burundi | 2016-17 | 204 | 5.1 |
| Congo DR. | 2013-14 | 541 | 13.6 |
| Congo | 2011-12 | 384 | 17.8 |
| Côte d'Ivoire | 2011-12 | 194 | 9.7 |
| Cameroon | 2011 | 170 | 4.7 |
| Ethiopia | 2016 | 661 | 18.9 |
| Gabon | 2012 | 233 | 12.7 |
| Ghana | 2014 | 66 | 3.4 |
| Gambia | 2013 | 17 | 0.7 |
| Guinea | 2018 | 677 | 26.4 |
| Kenya | 2014 | 215 | 3.5 |
| Comoros | 2012 | 142 | 11.0 |
| Liberia | 2013 | 278 | 14.5 |
| Lesotho | 2014 | 48 | 3.1 |
| Mali | 2018 | 888 | 40.2 |
| Malawi | 2015-16 | 1181 | 22.4 |
| Mozambique | 2015 | 346 | 22.3 |
| Nigeria | 2018 | 731 | 8.7 |
| Niger | 2012 | 316 | 16.6 |
| Namibia | 2013 | 92 | 5.0 |
| Rwanda | 2014-15 | 41 | 1.5 |
| Sierra Leone | 2013 | 411 | 10.1 |
| Senegal | 2010-11 | 369 | 10.2 |
| South Africa | 2016 | 38 | 2.5 |
| Chad | 2014-15 | 86 | 2.2 |
| Togo | 2013-14 | 127 | 7.3 |
| Uganda | 2016 | 867 | 20.3 |
| Zambia | 2013-14 | 550 | 14.9 |
| Zimbabwe | 2015 | 379 | 17.6 |
| Total |  | 11,474 | 100.0 |

## Study variables

### Dependent variable

The dependent variable employed for this study was “contraceptive use” which was derived from ‘current contraceptive method’. The responses were coded 0 = “No method”, 1 = “folkloric method”, 2= “traditional method” and 3 = “modern method”. The existing DHS variable excluded women who were pregnant and those who had never had sex. The modern methods included female sterilization, male sterilization, intrauterine contraceptive device

(IUD), contraceptive injection, contraceptive implants (Norplant), contraceptive pill, condoms, emergency contraception, standard day method (SDM), vaginal methods (foam, jelly, suppository), lactational amenorrhea method (LAM), country-specific modern methods and respondent-mentioned other modern contraceptive methods (including cervical cap, contraceptive sponge, and others). Periodic abstinence (rhythm, calendar method), withdrawal (coitus interruptus) and country-specific traditional methods of proven effectiveness were considered as traditional methods whiles locally described methods and spiritual methods of unproven effectiveness, such as herbs, amulets, gris-gris, etc. were the folkloric methods. To obtain a binary outcome, all respondents who said they used ‘no method’ were put in one category and were given the code ‘0 = No’ whereas those who were using either folkloric, traditional or modern method were also put into one category and given the code ‘1= Yes’.

### Explanatory variables

The main explanatory variable was reproductive health decision-making capacity. Two derivative variables that focused on decision-making on sexual intercourse and condom use were done. For the decision-making on sexual intercourse, female adolescents were asked if they can refuse their partners sex while for the decision-making on condom use, female adolescents were asked if they can ask their partners to use condoms during sexual intercourse. Reproductive health decision-making capacity was then generated from the combination of the decision-making on sexual intercourse and condom use variables. Following previous studies on reproductive health decision-making capacity [18,19], the original response category of these variables (1 = yes, 2 = no and 3 = don’t know/ not sure) were re-categorised whereby “no and don’t know/not sure” were recoded as “0” with “yes” recoded as 1. Responses coded as “0” were considered as not capable in making reproductive health decision whilst those coded as “1" were labelled as capable of making reproductive health decisions.

Apart from the main explanatory variable, eight other variables were considered in our study as covariates. These variables were age, place of residence, wealth quintile, occupation, educational level, age at first sex, and survey country. Again, apart from survey country, these variables were not determined a priori, instead their selection was based on their significant association with the outcome variable–contraceptives use, as reported in previous studies [27,28]. Four of the covariates were recoded to make them meaningful for analysis and interpretation. Marital status was recoded into ‘cohabiting (1)’ and ‘married (2)’. Occupation was captured as ‘not working (0)’ and ‘working (1)’. Educational level was recoded as ‘no education (0)’, ‘primary (1)’ and ‘secondary/higher (2)’. Age at first sex was coded as ‘less than 16 years (1)’and ‘16-19 years (2)’.

## Statistical analysis

The analysis begun with computation of contraceptive prevalence and proportion of female adolescents who could refuse a partner sex, ask a partner to use condom during sex and those who have the capacity to make reproductive health decisions in the 32 SSA countries.

The syntax “metan” in STATA version 14.0 (StataCorp, College Station, TX, USA) was used to generate forest plots for each of these indicators. Each forest plot showed the prevalence of an indicator in individual countries and its corresponding weight, as well as the pooled prevalence in all the countries and its associated 95% confidence intervals (CI). A test of heterogeneity of the DHS data obtained for the different countries showed a high level of inconsistency (I^2^ > 50%) thereby warranting the use of a random effect model in all the metaanalysis (see Figures 1, 2, 3 and 4). Secondly, we appended the datasets and generated a total sample of 11,474. After appending, we calculated contraceptive prevalence across the sociodemographic characteristics with their significance levels and chi square values [χ^2^]. Multicollinearity test was performed and with a mean VIF of 1.21, there was no evidence of multicollinearity between the variables (see S1 Table). Using the explanatory variables which were significantly associated with contraceptive use (p<0.05) among female adolescents from the chi-square test, binary logistic regression analysis was then carried out in a hierarchical order where Model I looked at a bivariate analysis of the main independent variable

[reproductive health decision-making capacity] and the outcome variable [contraceptive use]. Model II, on the other hand, looked at a complete model of all the explanatory variables and the outcome variable [see Table 3]. In line with research evidence that modern contraceptives are the most effective contraceptives [29,30,31], a further analysis was done to examine the influence of reproductive health decision-making capacity on modern contraceptive use (see

Table 4). All frequency distributions were weighted while the survey [svy] command in STATA version 14.2 was used to adjust for the complex sampling structure of the data in the regression analyses. Results for the regression analysis have been presented as Crude Odds Ratios [COR] and Adjusted Odds Ratios (AOR) with their corresponding 95% confidence intervals [CI] signifying precision and significance of the reported OR values. Statistical significance was declared at p<0.05.

## Ethical Approval

The DHS surveys obtain ethical clearance from the Ethics Committee of ORC Macro Inc. as well as Ethics Boards of partner organizations of the various countries such the Ministries of Health. During each of the surveys, either written or verbal consent was provided by the women. Since the data was not collected by the authors of this manuscript, official permission was sought from MEASURE DHS website and access to the data was provided after our intent for the request was assessed and approved on 3rd April, 2019. Data is available on [https://dhsprogram.com/data/available-datasets.cfm.](https://dhsprogram.com/data/available-datasets.cfm)

# Results

## Percentage scores on the prevalence of contraceptive use within selected SSA countries

The prevalence of contraceptive use in each of the 32 SSA countries included in the study are presented in Figure 1. The overall prevalence of contraceptive use was 20.07% (95% CI: 16.1424.01), ranging from 2.30% (95% CI:0.87-5.47) in Chad to 48.8% (95% CI: 33.50-64.10) in Rwanda.

**Fig 1: Contraceptive prevalence among female adolescents in SSA**

### Proportion of female adolescents who can refuse sex to a partner

Figure 2 presents results on the proportion of female adolescents in SSA who could refuse sex to their partners. The results indicate that 62.25% (95% CI=55.75-68.76) of female adolescents could refuse sex to their partners in SSA. Majority of female adolescents who could refuse their partners sex were in Cameroon [62.25% (95% CI=55.75-68.76)] whiles the smallest proportion was in Senegal [27% (95% CI=22.47-31.53)].

### Fig 2: Proportion of female adolescents in SSA who can refuse their partners sex Proportion of female adolescents who can ask their partners to use condom during sex

Figure 3 presents results on the proportion of female adolescents in SSA who could ask their partners to use condom during sex. Overall, 56.95% (95% CI=48.47-65.42) of female adolescents could ask their partners to use condom during sex in SSA. Out of this number, majority of them were in Namibia [91.30% (95% CI=85-54-97.06)] whiles a few of them were in Niger [18% (95% CI=13.76-22.24)].

**Fig 3: Proportion of female adolescents in SSA who can ask their partners to use condom during sex**

### Proportion of female adolescents in SSA who have the capacity to make reproductive health decisions

Figure 4 presents results on the proportion of female adolescents in SSA who have the capacity to make reproductive health decisions. Overall, 71.97% (95% CI: 65.48-78.46) of female adolescents in all the 32 SSA countries had the capacity to make reproductive health decisions.

Female adolescents in Niger had the lowest reproductive health decision-making capacity [32.90% (95% CI: 27.72-38.08] whiles those in Lesotho had the highest proportion of reproductive health decision-making capacity [95.80% (95% CI: 90.13-101.47].

**Fig 4: Proportion of female adolescents in SSA who have the capacity to make reproductive health decisions**

## Chi-square results on contraceptive use across socio-demographic characteristics

Table 2 provides a summary the proportion of contraceptive use across the selected sociodemographic characteristics. Nineteen-year-old adolescents had a greater proportion of contraceptive use [25.3%]. Contraceptive use was high among female adolescents in urban areas [24.2%] compared to those in rural areas [17.9%]. With wealth quintile, female adolescents with the richest wealth quintile had the highest proportion of contraceptive use [28.1%]. In terms of occupation, female adolescents who were working had the highest contraceptive prevalence [20.1%]. Female adolescents who were cohabiting recorded the highest proportion of contraceptive use [22.6%] compared to those who were married [18.2%]. Female adolescents with secondary/higher education [28.0%] and those whose age at first sex ranged between 16 and 19 years [20.6%] had the highest proportion of contraceptive use with respect to education level and age at first sex respectively. Apart from occupation, all the explanatory variables showed statistically significant associations with contraceptive use among female adolescents at 95% confidence interval (see Table 2).

### Table 2: Socio-demographic characteristics and contraceptive use among female adolescents in SSA

| **Variables** | **Weighted N** | **Weighted %** | **Contraceptive use** | |
| --- | --- | --- | --- | --- |
| **Age [***χ^2^*=182.3, p<0.001] |  |  | **N** | **%** |
| 15 | 477 | 4.2 | 44 | 8.5 |
| 16 | 1,067 | 9.3 | 129 | 11.8 |
| 17 | 1,935 | 16.9 | 319 | 16.6 |
| 18 | 3,836 | 33.3 | 706 | 18.6 |
| 19 | 4,169 | 36.3 | 1,050 | 25.3 |
| **Residence [***χ^2^*=56.0, p<0.001] |  |  |  |  |
| Urban | 3,011 | 26.2 | 733 | 24.2 |
| Rural | 8,463 | 73.8 | 1,515 | 17.9 |
| **Wealth Quintile [***χ^2^*=101.0, p<0.001] | |  |  |  |
| Poorest | 2,797 | 24.4 | 501 | 16.0 |
| Poorer | 2,856 | 24.9 | 505 | 17.7 |
| Middle | 2,528 | 22.0 | 463 | 19.5 |
| Richer | 2,023 | 17.6 | 433 | 22.9 |
| Richest | 1,270 | 11.1 | 346 | 28.1 |
| **Occupation [***χ^2^*=1.8, p>0.05] |  |  |  |  |
| Not working | 5,040 | 43.9 | 1,063 | 19.1 |
| Working | 6,434 | 56.1 | 1,185 | 20.1 |
| **Marital Status [***χ^2^*=30.3, p<0.001] | |  |  |  |
| Married | 7,977 | 69.5 | 1,429 | 18.2 |
| Cohabiting | 3,497 | 30.5 | 819 | 22.6 |
| **Educational Level [***χ^2^*=545.5, p<0.001] | |  |  |  |
| No Education | 3,389 | 29.5 | 230 | 6.7 |
| Primary | 4,906 | 42.8 | 1,167 | 23.4 |
| Secondary | 3,178 | 27.7 | 851 | 28.0 |
| **Age at First Sex [***χ^2^*=5.5, p<0.05] | |  |  |  |
| Less than 16 years | 6,395 | 55.7 | 1,233 | 18.8 |
| 16-19 years | 5,079 | 44.3 | 1,015 | 20.6 |

Source: Authors’ computations

## Binary Logistic regression analysis on female adolescents’ health decision-making capacity and contraceptive use

Results in Table 3 on reproductive health decision-making capacity and contraceptive use showed that female adolescents who had the capacity to make reproductive health decisions were more likely to use contraceptives [OR = 2.41; CI = 2.15 – 2.71, p < 0.001] compared to those who did not have the capacity to make reproductive health decision, even after controlling for the effect of the covariates [AOR = 1.41; CI = 1.24–1.60, p < 0.001]. Compared to Chad, female adolescents in Zimbabwe had the highest odds of using contraceptives [AOR = 36.50; CI = 8.98–148.3, p < 0.001]. The odds of contraceptive use among female adolescents increased with age, with those aged 19 years having the highest likelihood of using contraceptives [AOR = 3.59; CI = 2.56–5.04, p < 0.001]. The results further showed that the higher level of education was strongly linked to the high contraceptive use among female adolescents and this was more predominant among individuals with secondary education [AOR = 2.46; CI = 2.04–2.98, p < 0.001].

Female adolescents who were cohabiting had higher odds of using contraceptives compared to those who were married [AOR = 1.57; CI = 1.34–1.83, p < 0.001]. The odds of contraceptive use was highest among female adolescents with richest wealth quintile compared to those with poorest wealth quintile [AOR = 1.51; CI = 1.22–1.87, p < 0.001]. Female adolescents in rural areas were less likely to use contraceptives compared to those in urban areas [AOR = 0.77; CI = 0.67–0.89, p < 0.001]. Regarding age at first sex, female adolescents whose age at first sex was between 16 and 19 years were less likely to use contraceptives compared to those whose age at first sex was less than 16 years [[AOR = 0.64; CI = 0.58–0.72], p < 0.001](see Table 3).

### Table 3: Multivariable analysis on determinants of contraceptive use among female adolescents in SSA

| **Variables** | **Model I**  **COR[95%CI]** | **Model II**  **AOR[95%CI]** |
| --- | --- | --- |
| **Reproductive health decision making capacity** |  |  |
| Incapable | Ref | Ref |
| Capable | 2.41^***^[2.15–2.71] | 1.41^***^[1.24–1.60] |
| **Country** |  |  |
| Angola |  | 1.92 [0.46–7.99] |
| Burkina Faso |  | 7.09^**^ [1.70–29.5] |
| Benin |  | 3.46 [0.80–14.99] |
| Burundi |  | 11.13^***^ [2.68–46.26] |
| Congo Dr. |  | 3.98 [0.97–16.33] |
| Congo |  | 15.60^***^ [3.81–63.87] |
| Côte d'Ivoire |  | 7.02^**^ [1.65–29.80] |
| Cameroon |  | 7.90^**^ [1.88–33.14] |
| Ethiopia |  | 18.58^***^ [4.62–74.76] |
| Gabon |  | 8.99^**^ [2.16–37.43] |
| Ghana |  | 7.98^**^ [1.78–35.87] |
| Gambia |  | 5.15 [0.72–36.63] |
| Guinea |  | 5.88^*^ [1.44–24.03] |
| Kenya |  | 17.52^***^[4.26–72.18] |
| Comoros |  | 10.69^**^[2.53–45.25] |
| Liberia |  | 2.94 [0.69–12.46] |

| Lesotho |  | 17.21^***^[3.83–77.33] |
| --- | --- | --- |
| Mali |  | 6.66^**^ [1.64–26.98] |
| Malawi |  | 30.52^***^[7.62–122.2] |
| Mozambique |  | 6.16^*^ [1.50–25.34] |
| Nigeria |  | 2.95 [0.71–12.25] |
| Niger |  | 8.26^**^ [1.97–34.62] |
| Namibia |  | 16.46^***^ [3.80–71.29] |
| Rwanda |  | 22.72^***^ [4.92–105.0] |
| Sierra Leone |  | 3.96 [0.96–16.42] |
| Senegal |  | 4.51^*^ [1.05–19.28] |
| Chad |  | Ref |
| Togo |  | 3.93 [0.87–17.64] |
| Uganda |  | 8.21^**^ [2.03–33.23] |
| South Africa |  | 28.29^***^ [6.02–132.9] |
| Zambia |  | 22.12^***^ [5.48–89.27] |
| Zimbabwe |  | 36.50^***^[8.98–148.3] |
| **Age** |  |  |
| 15 |  | Ref |
| 16 |  | 1.44[0.99–2.09] |
| 17 |  | 2.27^***^[1.59–3.22] |
| 18 |  | 2.80^**^[1.99–3.93] |
| 19 |  | 3.59^***^[2.56–5.04] |
| **Place of residence** |  |  |
| Urban |  | Ref |
| Rural |  | 0.77^***^[0.67–0.89] |
| **Educational level** |  |  |
| No Education |  | Ref |
| Primary |  | 1.97^***^[1.66–2.34] |
| Secondary/Higher |  | 2.46^***^[2.04–2.98] |
| **Marital status** |  |  |
| Married |  | Ref |
| Cohabiting |  | 1.57^***^[1.34–1.83] |
| **Wealth quintile** |  |  |
| Poorest |  | Ref |
| Poorer |  | 1.12[0.96–1.29] |
| Middle |  | 1.21^*^[1.04 –1.41] |
| Richer |  | 1.34^**^[1.13–1.60] |
| Richest |  | 1.51^***^[1.22–1.87] |
| **Age at First Sex** |  |  |
| Less than 16 years |  | Ref |
| 16-19 years |  | 0.64^***^[0.58–0.72] |
| *N* | 11,474 | 11,474 |
| pseudo *R*^2^ | 0.023 | 0.155 |

314

315 * p<0.05, ** p<0.01, *** p<0.001; Ref=Reference , CI=Confidence Intervals , COR=Crude
316 Odds Ratio, AOR=Adjusted Odds Ratio
317 Source: Authors’ computations

## Binary Logistic regression analysis on female adolescents’ health decision-making capacity and modern contraceptive use

Results in Table 4 on reproductive health decision-making capacity and modern contraceptive use showed that female adolescents who had the capacity to make reproductive health decisions were more likely to use modern contraceptives [OR = 2.29; CI = 2.03 – 2.58], p < 0.001] compared to those who did not have the capacity to make reproductive health decision, even after controlling for the effect of the covariates [AOR = 1.38; CI = 1.21–1.58], p < 0.001]. Compared to Chad, the highest odds of modern contraceptives use was among female adolescents in Zimbabwe [AOR = 34.97; CI = 8.61–142.1, p < 0.001]. The likelihood of modern contraceptive use among female adolescents increased with age, with those aged 19 years having the highest likelihood of using contraceptives [AOR = 3.24; CI = 2.26–4.63, p < 0.001]. The odds of modern contraceptives usage were low among female adolescents in rural areas compared to those in urban areas [AOR = 0.72; CI = 0.63–0.84, p < 0.001]. The results further showed that the higher the level of education, the more likely some female adolescents will use modern contraceptives and this was more predominant among individuals with secondary education [AOR = 2.49; CI = 2.03–3.05, p < 0.001].

Female adolescents who were cohabiting were more likely to use modern

contraceptives compared to those who were married [AOR = 1.48; CI = 1.26–1.75, p < 0.001]. The odds of modern contraceptive use was highest among female adolescents with richest wealth quintile compared to those with poorest wealth quintile [AOR = 1.45; CI = 1.16–1.81], p < 0.001]. Regarding age at first sex, female adolescents whose age at first sex was between 16 and 19 years were less likely to use modern contraceptives compared to those whose age at first sex was less than 16 years [AOR = 0.63; CI = 0.58–0.71, p < 0.001](see Table 4).

342	**Table 4: Multivariable analysis on determinants of modern contraceptive use among**

### 343 female adolescents in SSA

| **Variables** | **Model I**  **COR[95%CI]** | **Model II**  **AOR[95%CI]** |
| --- | --- | --- |
| **Reproductive health decision making capacity** |  |  |
| Incapable | Ref | Ref |
| Capable | 2.29^***^ [2.03–2.58] | 1.38^***^ [1.21–1.58] |
| **Country** |  |  |
| Angola |  | 1.76 [0.42–7.36] |
| Burkina Faso |  | 6.403^*^[1.53–26.74] |
| Benin |  | 2.82 [0.64–12.37] |
| Burundi |  | 9.42^**^ [2.26–39.37] |
| Congo Dr. |  | 1.27 [0.30–5.43] |
| Congo |  | 7.19^**^[1.74–29.64] |
| Côte d'Ivoire |  | 3.17 [0.71–14.16] |
| Cameroon |  | 4.98^*^[1.17–21.18] |
| Ethiopia |  | 17.98^***^[4.47–72.32] |
| Gabon |  | 5.48^*^ [1.30–23.04] |
| Ghana |  | 6.70^*^ [1.47–30.54] |
| Gambia |  | 4.94 [0.69–35.29] |
| Guinea |  | 5.53^*^ [1.35–22.61] |
| Kenya |  | 16.04^***^[3.89–66.12] |
| Comoros |  | 6.61^*^ [1.53–28.50] |
| Liberia |  | 3.00 [0.71–12.73] |
| Lesotho |  | 17.10^***^ [3.81–76.80] |
| Mali |  | 5.89^*^[1.45–23.88] |
| Malawi |  | 29.75^***^[7.43–119.2] |
| Mozambique |  | 5.91^*^ [1.43–24.32] |
| Nigeria |  | 1.96 [0.47–8.29] |
| Niger |  | 7.47^**^ [1.78–31.37] |
| Namibia |  | 15.14^***^[3.49–65.74] |
| Rwanda |  | 23.98^***^[5.18–110.9] |
| Sierra Leone |  | 3.94[0.95–16.34] |
| Senegal |  | 3.44 [0.79–14.92] |
| Chad |  | Ref |
| Togo |  | 3.29 [0.72–15.07] |
| Uganda |  | 7.75^**^[1.91–31.39] |
| South Africa |  | 28.09^***^[5.97–132.1] |
| Zambia |  | 19.70^***^[4.88–79.51] |
| Zimbabwe |  | 34.97^***^[8.61–142.1] |
| **Age** |  |  |
| 15 |  | Ref |
| 16 |  | 1.37 [0.92–2.04] |
| 17 |  | 2.10^***^[1.44–3.04] |
| 18 |  | 2.51^***^[1.75–3.60] |

| 19 |  | 3.24^***^[2.26–4.63] |
| --- | --- | --- |
| **Place of residence** |  |  |
| Urban |  | Ref |
| Rural |  | 0.72^***^[0.63–0.84] |
| **Educational level** |  |  |
| No Education |  | Ref |
| Primary |  | 1.99^***^[1.65–2.39] |
| Secondary/Higher |  | 2.49^***^[2.03–3.05] |
| **Marital status** |  |  |
| Married |  | Ref |
| Cohabiting |  | 1.48^***^[1.26–1.75] |
| **Wealth quintile** |  |  |
| Poorest |  | Ref |
| Poorer |  | 1.10 [0.94–1.29] |
| Middle |  | 1.21^*^[1.03–1.42] |
| Richer |  | 1.30^**^[1.08–1.56] |
| Richest |  | 1.45^***^[1.16–1.81] |
| **Age at First Sex** |  |  |
| Less than 16 years |  | Ref |
| 16-19 years |  | 0.63^***^[0.58–0.71] |
| *N* | 11,474 | 11,474 |
| pseudo *R*^2^ | 0.02 | 0.164 |

* p<0.05, ** p<0.01, *** p<0.001; Ref=Reference , CI=Confidence Intervals , COR=Crude

Odds Ratio, AOR=Adjusted Odds Ratio

Source: Authors’ computations

# Discussions

This multi-country study assessed the influence of female adolescents’ reproductive health decision-making capacity on their contraceptive usage in 32 SSA. The study was critical since female’s ability to take decisive reproductive health decisions including choices in contraceptive use (i.e., condom among others) can lead to good reproductive health [19] and also the prevention of certain sexually transmittable infections (e.g. HIV, STI’s and Hepatitis among others) [26]. Generally, 71.97% of females across the 32 countries included in the study had the capacity (i.e., empowerment) to make reproductive health decisions, a finding that is similar but higher compared to 69.3% capacity found in other studies (e.g., [32]). This variation might be due to differences in the study populations and the number of countries these studies were based on. We found that females who had capacity to make reproductive health decisions were more likely to use contraceptives. This does not necessarily imply that females who had no capacity (i.e. empowerment) had no priority of contraceptive use. Some scholars have found that females’ restrictive roles in domestic matters including reproductive health issues in many sub-Saharan countries (e.g., Ghana, Kenya, Tanzania, Nigeria, Uganda and South Africa) rest with men [33], a reason that could derail them from contraceptive usage. Female adolescents from Zimbabwe had the highest odds of contraceptive usage compared to Angola with the least. This results is not surprising because Zimbabwe, together with other southern African states (e.g., Namibia) have one of the most effective family planning programs in sub-Saharan Africa (SSA) with lowest unmet need for contraception among adolescent women in Africa [34]. Other plausible reasons might be Zimbabwe’s positive success in female adolescents’ contraceptive usage and their high literacy rate imply that their adolescents might have capacity in many other areas of life such as socio-cultural and familial empowerment [35] compared to other young females in the other 31 SSA countries. What may also be accounting for these sub-Saharan societies with lower odds of using contraceptives could be ascribed to the low acceptance and high cultural resistance to family planning. According to [36], the social, financial, and strict kingship norms and values devoted to children and family in the region are also contributory toward the application of contraceptives.

An age progression in contraceptive usage, with those aged 19 years having the highest likelihood of using contraceptives in the current study. This finding corroborate previous studies in developing countries like Pakistan [37], Nepal [38] and Ghana [19] where females’ reproductive health decision making capacity increase with age as their other dimensions of empowerment increases. Female adolescents in rural dwellings were less likely to use contraceptives compared to their urban counterparts. This disparity in rural-urban contraceptive use could be due to limited and lack of access to health care services in rural communities, and even where they exist, socio-cultural limitations may hinder usage [39]. This assertion reinforces the rural-urban inequities in terms of essential services like reproductive health (i.e., family planning services) for female adolescents. This finding can best be appreciated when we reflect on how health facilities, key human and other resources are skewed in favour of urban settings in most countries across SSA [40]. African governments and non-governmental organisations could roll out reproductive health services, the adoption, and replication of Community Health Programme Services (CHPS) concept across the subregion to grant quality healthcare access to rural dwellers.

The higher the level of education, the more likely some female adolescents will use contraceptives, predominantly among those with secondary/higher education. Specifically, the odds of contraceptive use were higher for those with secondary and/ or higher education, compared with those with none, a finding similar to previous studies in Ghana and other low and middle-income countries [41,18]. This finding strongly implies that educating girls may be an effective tool for promoting reproductive health decision making capacity and contraceptive use. Educated females adolescents may have better access to health care information and reproductive health literature, better ability to use quality healthcare services and greater autonomy to make decisions [42]. Other studies have also proven that education may impart feelings of self-worth and self-confidence, which are necessary for changing health behaviour and seeking out health services for the first time [43].

The odds of contraceptive use were highest among female adolescents with richest wealth quintile compared to those with poorest wealth quintile. Consistent with previous studies in Africa [27] and elsewhere [44] that found women with high wealth index to have high affinity for contraceptive use and use of contraceptives. For example, [45] explained that

Ghanaian women who have high wealth status are more capable to take charge of their sexual and reproductive health matters (i.e. contraceptive use) than women of poorer wealth status. It is sound to argue that rich women who are but few in Africa, gain more repute, boost their confidence and are self-reliant to independently decide and afford any contraceptive of their choice. However, it is fair to extrapolate that females’ adolescents from the poorest wealth quintile may need to be more sexually empowered than their richer counterparts to overcome the greatest barriers accounting for the utilization of health services, including contraceptives.

African governments and gender empowerment NGO’s within the sub-region should target much of their social interventions (i.e. pro-poor) programs toward adolescent females, as most women in Africa are below the poverty line (evidence). Specific competency-based and life, skills-based education training interventions should be implemented across the sub-region by governments to broaden the scope of female adolescents’ reproductive health issues [46].

## Practical implications

Female adolescents’ ability to make reproductive health decisions was associated with contraceptive use. Nonetheless, it is imperative to recognize that female adolescents’ involvement in positive reproductive decisions is contingent strongly on the shared or interactive influence of their age, rural-urban geographical location, educational status, and socio-economic status. For most parts of SSA, many decisions on managing sexual and reproductive health have come under serious scrutinized from a socio-cultural lens as female adolescents’ vulnerability to unintended pregnancy, unsafe abortions, HIV and other STI’s have increased. For example, a deep rooted cultural barrier to contraceptive use in many parts of SSA could be the unfriendliness that many female adolescents experience when they visit maternal and child health clinics and other primary sources of contraceptives. Due to overt social dissatisfaction of premarital sexual activity and the general lack of confidentiality, and anonymity at these health centres, many young girls, by attempting to procure contraceptives, are exposed to public ridicule, gossip and negative attitudes from health personnel [47]. Therefore, female adolescents with lower reproductive health decision making capacity could be at high risk of mistreatment from health care providers, either by getting poor-quality services, or suspending health services all together [43].

Education, particularly secondary and/ or higher education, has repetitively been shown to be connected with a wide range of positive sexual reproductive health outcomes such as contraceptive use, age of marriage, number of births, and use of health services, albeit other proxies (e.g., socio-economic status). One systematic review of risk and protective factors for ASRH in low- and middle-income countries found that the more current in-school adolescents spend in school, the less likely they would ever engage in sex and have greater chances of using modern contraceptives compared to those who leave school early [48,49]. Other studies have proven that education may impart feelings of self-worth and self-confidence, which are necessary for changing health behaviour and seeking out health services for the first time [47]. Therefore, educational intervention programs that would promote the enrolment and retention of female adolescents in secondary schools and perhaps beyond are critical toward improving female adolescents’ reproductive health decision making capacity and their overall health.

Undoubtedly, though the DHS includes standard questions that allow multi-country comparisons, further in-depth qualitative enquiry and statistical linkages between different components of reproductive health decision making and adolescents’ likelihood to engage in sexual behaviours (e.g., onset of sexual debut, unsafe abortions, unintended pregnancies) would be helpful to better understand the context specific issues across sub-Sahara Africa. Critical consideration must be given to context specific institutional barriers (e.g., cultural norms and expectations for sex roles) to adolescents’ sexuality such as pregnancy prevention, healthcare providers' attitudes, and lack of confidentiality, and their impact on adolescents' capacity to make decisions about sexual and reproductive health issues [17].

## Strengths and Limitations

The main strength of the study is the use of national representative surveys whose methodology has been validated across the countries and as a result make the findings of our study valid and generalizable to other adolescents in SSA. Despite these strengths, the current study is not without limitations. First, the DHS data presented in this study is limited to 32 SSA countries and therefore may not be applicable to other countries and geographical settings. Second, this study is linked to the cross-sectional nature of DHS data. Given that the data for women's empowerment and contraceptive use were collected within a specific time frame, causal inferences or temporal relationship between studied variables cannot be ascertained. There is a probability that the similar observed or unobserved factors may impact both female adolescents’ reproductive health decision making capacity and contraceptive method used. For the purpose of robustness, some factors that are theoretically associated to contraceptive use but not to empowerment were statistically controlled, hence full testing and adjusting for endogeneity is necessary, but was beyond the scope of this study. Respondent needed to have answered ‘yes’ to both “ ability to refuse partners sex and ability to ask partner to use condom in order to be considered to have ‘reproductive health decision-making capacity. With this women using another contraceptive method might find it strange or difficult to request that their partner, particularly in longer-term or more serious relationships, use a condom (double protection). Second, the outcome variable, of contraceptive use is limited insufficiently nuanced to consider whether the adolescent has a need to avoid pregnancy. Also, the current study used the most recent DHS data, hence, variations in contraceptive accessibility and usage may have happened in the last few years. For example, countries like Zimbabwe and Namibia, have been promoting long-standing family planning services in some of their districts, whereas Zambia has seen a drastic shift in funding from family planning to HIV prevention and management. While these alterations are not likely to have caused changes in contraceptive use within a specific time frame, possible long term changes in these policy shifts may lead to increased usage of specific contraceptive methods (e.g., condom use) at the expense of others (i.e., IUD). Therefore, there may also be variations with the linkages between reproductive health decision making capacity and contraceptive use identified in this [42]. Lastly, due to the very sensitive nature of the sexuality themes under consideration, there is the possibility of social desirability concerns or recall bias among the study participants.

# Conclusions

Utilization of contraceptives remains low in SSA among adolescents. This study revealed that reproductive health decision making capacity, age, education, wealth status, marital status, place of residence and age at first sex are associated with contraceptives use. It is therefore imperative to strengthen existing efforts in SSA on contraceptives use among adolescent by empowering adolescents to take reproductive health decisions, targeting younger adolescents, those in rural areas, those without formal education and those who initiate sexual intercourse between aged 16 and 19 years. These can help reduce teenage pregnancy and early childbirth, reduce maternal mortality and help in the achievement of SDG three. The current findings suggest that specific programs that aim to promote female adolescent's capacity to discuss sexual activity would be particularly be helpful in SSA countries (e.g., Chad, Gambia) that were identified with the lower odds of using contraceptives and where family planning services are limited. These programs should complement efforts to increase contraceptives’ availability to meet the rising demand for modern methods due to serious child and maternal health issues.

## Declaration of Conflicting Interests

The authors declared no potential conflicts of interest with the research, authorship, and/or publication of this article.

## Funding

The authors received no financial support for the research, authorship, and/or publication of this article.

# References

1. Blackstone SR, Nwaozuru U, Iwelunmor J. Factors influencing contraceptive use in subSaharan Africa: a systematic review. International quarterly of community health education. 2017 Jan; 37(2):79-91.
2. Doyle AM, Mavedzenge SN, Plummer ML, Ross DA. The sexual behaviour of adolescents in sub Saharan Africa: patterns and trends from national surveys. Tropical Medicine & International Health. 2012 Jul;17(7):796-807.
3. Chandra-Mouli, Venkatraman, Donna R. McCarraher, Sharon J. Phillips, Nancy E.

Williamson, and Gwyn Hainsworth. “Contraception for adolescents in low and middle income countries: Needs, barriers, and access,” Reproductive Health. 2014:11(1): 1–8. [https://doi.org/10.1186/1742-4755-11-1.](https://doi.org/10.1186/1742-4755-11-1)

4. Radovich, Emma, Mardieh L. Dennis, Kerry L.M. Wong, Moazzam Ali, Caroline A. Lynch, John Cleland, Onikepe Owolabi, Mark Lyons-Amos, and Lenka Benova. “Who meets the contraceptive needs of young women in Sub-Saharan Africa?” Journal of

Adolescent	Health.	2018:62(3):273–280.

[https://doi.org/10.1016/j.jadohealth.2017.09.013.](https://doi.org/10.1016/j.jadohealth.2017.09.013)

5. Tsui AO, Brown W, Li Q. Contraceptive practice in sub-Saharan Africa. Population and development review. 2017 May; 43(Suppl Suppl 1):166.
6. Bundy DA, de Silva N, Horton S, Patton GC, Schultz L, Jamison DT, ... & Appleby L. Investment in child and adolescent health and development: key messages from Disease Control Priorities. *The Lancet*. 2018:*391*(10121), 687-699.

7. MacQuarrie, Kerry L.D. “Unmet Need for Family Planning Among Young Women:

Levels and Trends [CR34].” Rockville, Maryland. 2014.

8. Presser HB. Demography, feminism, and the science-policy nexus. *Population and Development Review*. 1997:295-331.
9. Mason KO & Smith HL. Women’s empowerment and social context: Results from five Asian countries. *Gender and Development Group, World Bank, Washington, DC*. 2003
10. Kim JC, Watts CH. Gaining a foothold: tackling poverty, gender inequality, and HIV in Africa. Bmj. 2005 Sep 29;331(7519):769-72.
11. Blanc AK & Wolff B. Gender and decision-making over condom use in two districts in Uganda. *African Journal of Reproductive Health*. 2001:15-28.
12. Achan SF, Akweongo P, Debpuur C, Cleland J. Coping strategies of young mothers at risk of HIV/AIDS in the Kassena-Nankana district of Northern Ghana. African Journal of Reproductive Health. 2009;13(1).
13. Barnett B, Konaté M, Mhloyi M, Mutambirwa J, Francis-Chizororo M, Taruberekera N, Ulin P. The impact of family planning on women's lives: Findings from the Women's

Studies Project in Mali and Zimbabwe. African Journal of Reproductive Health.	1999;3(1):27-

14. Okal J, Stadler J, Ombidi W, Jao I, Luchters S, Temmerman M, Chersich MF. Secrecy, disclosure and accidental discovery: perspectives of diaphragm users in Mombasa,

Kenya. Culture, health & sexuality. 2008 Jan 1;10(1):13-26.

15. Blanc AK. The effect of power in sexual relationships on sexual and reproductive

health: an examination of the evidence. Studies in family planning. 2001	Sep;32(3):189-213.

16. Bankole A, Singh S. Couples' fertility and contraceptive decision-making in developing 570 countries: hearing the man's voice. International family planning perspectives. 1998 Mar 1:15-24.
17. Do M, Kurimoto N. Women's empowerment and choice of contraceptive methods in selected African countries. International perspectives on sexual and reproductive health. 2012 Mar 1:23-33.
18. Darteh EK, Doku DT, Esia-Donkoh K. Reproductive health decision-making among

Ghanaian women. Reproductive health. 2014;11(1):23. DC: FOCUS on Young Adults;

May 22.

19. Seidu AA, Ahinkorah BO, Agbemavi W, Amu H, Bonsu F. Reproductive health decision making capacity and pregnancy termination among Ghanaian women: Analysis of the 2014 Ghana demographic and health survey. Journal of Public Health. 2019:1-0.
20. Kulczycki A: The Abortion Debate in the World Arena. London: Macmillan Press Limited;

1999.

21. Oronje RN, Crichton J, Theobald S, Lithur NO, Ibisomi L, Oronje RN, Crichton J, Theobald S, Lithur NO, Ibisomi L: Operationalising sexual and reproductive and rights in sub-Saharan Africa: constraints, dilemmas and strategies. BMC Int Health Hum Rights 2011, 11(Suppl 3):S8.
22. UNFPA: ICPD 1994: Summary of the programme of action. Adv Physiol Educ 1994,

:1995.

23. Bankole A, Ahmed FH, Neema S, Ouedraogo C, Konyani S. Knowledge of correct condom use and consistency of use among adolescents in four countries in Sub-Saharan Africa.

African journal of reproductive health. 2007; 11(3):197.

24. Manlove J, Ikramullah E, Terry-Humen E. Condom use and consistency among male adolescents in the United States. Journal of adolescent health. 2008 Oct 1;43(4):325-

33.

25. Bearinger, Linda H., Renee E. Sieving, Jane Ferguson, and Vinit Sharma. “Global perspectives on the sexual and reproductive health of adolescents: Patterns, prevention, and potential,” The Lancet. 2007:369(9568): 1220–1231. https://doi.org/

10.1016/S0140-6736(07)60367-5.

26. Bankole A & Malarcher S. Removing barriers to adolescents' access to contraceptive information and services. *Studies in family planning*. 2010:*1*(2), 117-124.
27. Achana FS, Bawah AA, Jackson EF, Welaga P, Awine T, Asuo-Mante E, Oduro A, Awoonor-Williams JK, Phillips JF. Spatial and socio-demographic determinants of contraceptive use in the Upper East region of Ghana. Reproductive health. 2015 Dec;12(1):29.
28. Mandiwa C, Namondwe B, Makwinja A, Zamawe C. Factors associated with contraceptive use among young women in Malawi: analysis of the 2015–16 Malawi demographic and health survey data. Contraception and Reproductive Medicine. 2018 Dec;3(1):12.
29. Abiye, A. A., Fekede, B., Jemberie, A. M., Molla, B. A., Tolla, B. K., Tefera, B. S., ... & Kemal, H. S. (2019). Modern Contraceptive Use and Associated Factors among Reproductive Age Group Women in three Peri-Urban Communities in Central Ethiopia. *Journal of Drug Delivery and Therapeutics*, *9*(6-s), 93-102.
30. Ajayi, A. I., Adeniyi, O. V., & Akpan, W. (2018). Use of traditional and modern contraceptives among childbearing women: findings from a mixed methods study in two southwestern Nigerian states. *BMC public health*, *18*(1), 604.
31. Debebe, S., Limenih, M. A., & Biadgo, B. Modern contraceptive methods utilization and associated factors among reproductive aged women in rural Dembia District, northwest Ethiopia: Community based cross-sectional study. *International Journal of Reproductive BioMedicine*. 2017:*15*(6), 367.
32. Darteh EK, Dickson KS, Doku DT. Women’s reproductive health decision-making: A multi-country analysis of demographic and health surveys in sub-Saharan Africa. PloS one. 2019;14(1).
33. Tolhurst R, Amekudzi YP, Nyonator FK, Squire SB, Theobald S. “He will ask why the child gets sick so often”: The gendered dynamics of intra-household bargaining over healthcare for children with fever in the Volta Region of Ghana. Social Science & Medicine. 2008 Mar 1;66(5):1106-17.
34. Ngome E, Odimegwu C. The social context of adolescent women’s use of modern contraceptives in Zimbabwe: a multilevel analysis. Reproductive health. 2014 Dec;11(1):64.
35. Na M, Jennings L, Talegawkar SA, Ahmed S. Association between women’s empowerment and infant and child feeding practices in sub-Saharan Africa: an analysis of Demographic and health surveys. Public Health nutrition. 2015 Dec;18(17):3155-65.
36. Caldwell JC, Caldwell P. The cultural context of high fertility in sub-Saharan Africa.

Population and development review. 1987 Sep 1:409-37.

37. Saleem S, Bobak M. Women's autonomy, education and contraception use in Pakistan: a national study. Reproductive health. 2005 Dec;2(1):8.
38. Acharya DR, Bell JS, Simkhada P, Van Teijlingen ER, Regmi PR. Women's autonomy in household decision-making: a demographic study in Nepal. Reproductive health. 2010

Dec;7(1):15.

39. Muanda MF, Ndongo GP, Messina LJ, Bertrand JT. Barriers to modern contraceptive use in rural areas in DRC. Culture, health & sexuality. 2017 Sep 2;19(9):1011-23.
40. Yaya S, Bishwajit G, Gunawardena N. Socioeconomic factors associated with choice of delivery place among mothers: a population-based cross-sectional study in Guinea- Bissau. BMJ global health. 2019 Apr 1;4(2):e001341.
41. Atiglo DY, Codjoe SN. Meeting women’s demand for contraceptives in Ghana: Does autonomy matter?. Women & health. 2019 Apr 21;59(4):347-63.
42. Apanga PA, Adam MA. Factors influencing the uptake of family planning services in the Talensi District, Ghana. Pan African Medical Journal. 2015;20(1).
43. Singh, K., Bloom, S, Brodish, P. Gender equality as a means to improve maternal Social Sci Med 2013; 63: 2242-54.
44. Aslam SK, Zaheer S, Qureshi MS, Aslam SN, Shafique K. Socio-economic disparities in use of family planning methods among Pakistani women: findings from Pakistan demographic and health surveys. PloS one. 2016 Apr 7;11(4):e0153313.
45. Nyarko SH. Unintended Pregnancy among Pregnant Women in Ghana: Prevalence and Predictors. Journal of Pregnancy. 2019.
46. Ahinkorah BO, Hagan Junior JE, Seidu AA, Mintah JK, Sambah F, Schack T, Hormenu T. Examining Pregnancy Related Socio-Cultural Factors Among Adolescent Girls in the

Komenda-Edina-Eguafo-Abrem Municipality in the Central Region of Ghana: A Case Control Study. Frontiers in public health. 2019;7:93.

47. Senderowitz J. Health facility programs on reproductive health for young adults. Washington, DC: FOCUS on Young Adults; 1997 May 22.
48. Grown C, Gupta GR, Pande R. Taking action to improve women’s health through gender equality and women’s empowerment. The Lancet. 2005. Feb5;365(9458):541-3.
49. Mmari K, Sabherwal S. A review of risk and protective factors for adolescent sexual and reproductive health in developing countries: an update. Journal of Adolescent Health.

2013 Nov 1;53(5):562-72

Supporting Information

[Click here to access/download Supporting Information](https://www.editorialmanager.com/pone/download.aspx?id=26354723&guid=70e3ddf8-b7ab-4444-b63b-a9b362703476&scheme=1)

[Collinearity Diagnostics.docx](https://www.editorialmanager.com/pone/download.aspx?id=26354723&guid=70e3ddf8-b7ab-4444-b63b-a9b362703476&scheme=1)

Revised Manuscript with Track Changes

1	**Female adolescents’ reproductive health decision-making capacity**

## 2 and contraceptive use in sub-Saharan Africa: What does the future

3	**hold?**

4

5 Bright Opoku Ahinkorah^1^, John Elvis Hagan Jr.^2,4^, Abdul-Aziz Seidu^3^*, Francis Sambah^2^, 6 Faustina Adoboi^5^, Thomas Schack^4^, Eugene Budu^3^

7

8 ^1^The Australian Centre for Public and Population Health Research [ACPPHR], Faculty of
9 Health, University of Technology Sydney, Australia
10 ^2^Department of Health, Physical Education, and Recreation, University of Cape Coast, Cape 11	Coast, Ghana
12 ^3^Department of Population and Health, University of Cape Coast, Cape Coast, Ghana
13 ^4^Neurocognition and Action-Biomechanics-Research Group, Faculty of Psychology and
14 Sport Sciences, Bielefeld University, Bielefeld-Germany
15 ^5^Cape Coast Nursing and Midwifery Training College, Cape Coast, Ghana

16

### 17 *Corresponding author

18 Abdul-Aziz Seidu
19 Email: abdul-aziz.seidu@stu.ucc.edu.gh

20

21

22

23

24

# Abstract

## Introduction

Given the social, economic and health imports of early parenthood, unintended pregnancy, and the risks of HIV infection and subsequent transmission, there is an urgent need to understand how adolescents make sexual and reproductive decisions regarding contraceptive use. This study sought to assess the influence of female adolescents’ reproductive health decisionmaking capacity on their contraceptive usage.

## Materials and methods

Data was obtained from pooled current Demographic and Health Surveys [DHS] conducted in 32 countries in sub-Saharan Africa [SSA]. The unit of analysis for this study was sexually active female adolescents [n=11,474]. Bivariate and multivariable analyses were conducted using Pearson chi-square tests and binary logistic regression respectively. All analyses were performed using STATA version 14.2. Results were presented using Odds Ratios [OR] and adjusted Odds Ratios [AOR]. **Results**

The results showed that 71.9% of the adolescents had the capacity to make reproductive health decisions. It was also found that overall prevalence of contraceptive use was 20.07% and it ranged from 2.3% in Chad to 48.8% in Rwanda. In the multivariable analysis, adolescents who had the capacity to take reproductive health decisions had higher odds to use contraceptives [AOR = 1.41; CI = 1.24–1.60, p < 0.001]. Compared to Chad, female adolescents in Zimbabwe had the highest odds of using contraceptives [AOR = 36.50; CI = 8.98–148.3, p < 0.001]. The odds of contraceptive use among female adolescents increased with age, with those aged 19 years having the highest likelihood of using contraceptives [AOR = 3.59; CI = 2.56–5.04, p <

0.001]. The results further showed that the higher the level of education, the more likely some female adolescents will use contraceptives and this was more predominant among individuals with secondary education [AOR = 2.46; CI = 2.04–2.98, p < 0.001].

## Conclusion

Utilisation of contraceptives in general and modern contraceptives in particular remains low in among adolescents in SSA. Strengthening existing efforts in SSA on contraceptives usage among adolescents by empowering these young females to take positive reproductive health decisions is imperative. This enlightenment should target younger female adolescents, specifically, those in rural areas, and without formal education as well as those who initiate sexual intercourse between age 16 and 19 years. These can help reduce teenage pregnancy, prevent some infections, reduce maternal mortality and early childbirth and also help achieve SDG three.

**Keywords: Adolescents; Decision-making capacity; Contraceptives; Sub-Saharan**

**Africa**

# Introduction

Empirical data from sub-Sahara Africa (SSA) suggest that a high proportion of 15- to 19-yearolds are sexually active and at risk of contracting HIV, other STIs or of unplanned pregnancy because of lack of contraceptive use [1,2]. Although, recent statistics show that contraceptive use among young adult women in SSA has improved in line with global trends [3,4], young women in these age brackets disproportionately use contraceptives, especially short-term methods (e.g., condoms [4]). Given the heterogeneous nature of the SSA region, sub-regional variations exist in young adult women’s contraceptive use. Generally, among women of reproductive age (i.e., 15–49), reported modern contraceptive use between 2005 and 2015 is highest (54.3%) in Southern Africa (i.e., Botswana, Lesotho, Namibia, South Africa and

Swaziland), followed by Northern Africa (i.e., Algeria, Egypt, Libya, Morocco, Sudan, and Tunisia). The moderate to lowest trends are recorded in Eastern Africa (27.2%), Western (15%), and Central Africa (12%) countries [5]. According to Bundy et al. [6], it is possible that these developments could be similar for young adult populations across the region. This assertion explains why on the average, 26–30% of presently married women age 15–24 and 40–41% of recently unmarried women age 15–24 report an unmet need for family planning in the West/Central and East/South regions of Africa [7].

Scholarly information reveals that empowering women is generally seen to be critical for impacting family planning use and reducing HIV transmission [8,9,10]. Empowered women are often associated with the desire for fewer children, readily access more health services, avoid risky sexual partners, effectively strategize their births [10], and utilize condoms as HIV and STI prevention method [11,12]. Comparatively, enhanced health and contraceptive accessibility and usage may empower women [13]. For instance, women who accessed and utilized diaphragms applauded their recent aptitude to demonstrate safer sexual behaviour in Kenya [14]. Similarly, Blanc [15] showed that the steadiness of power in sexual relations has association with usage of health services and reproductive health outcomes. Despite a sizeable body of literature demonstrating the association between various dimensions of women empowerment on contraceptive usage, only few studies have explicitly measured the association between women health decision making capacity and contraceptive use in SSA [16,17,18,19]. Bankole and Singh (16) found that contraceptive usage was more pronounced when both couples decided to halt childbearing and reported lowest when both partners desired more children. Do and Kurimoto (17) later showed positive association between women empowerment score and contraceptive use in selected countries (i.e., Ghana, Namibia, Uganda, Zambia and Zimbabwe).

Despite these studies contributing to extant literature on women’s reproductive health decision making and additional knowledge to the limited information on sexual and reproductive health issues in SSA, some flaws should not be overlooked. Specifically, the National Demographic Survey data used by some of the researchers (e.g., Bankole and Singh; Do and Kurimoto) were compiled from different years and somewhat old, therefore may contain inconsistent variables with measurement errors and trends for effective analytical comparisons. Hence, findings from these studies may have been either over-exaggerated or under reported by the authors. Similarly, findings noted by Darteh et al., [18] and Seidu et al., [19] were very limited in scope (i.e., data from one country) and cannot be used for multicountry comparisons and make generalizations across SSA. Even more compelling for additional research is that, in many SSA countries, women’s right to health, including sexuality has been infringed because of socio-cultural barriers (e.g., most decisions taken by men, [20,21,22]).

Given the sparse literature on reproductive health decision making among adolescents in SSA, it is for strategic programmes and policy standpoint to better understand female adolescents’ health decision making capacity and contraceptive use behaviour for their later healthy sexual and reproductive lives [23,24]. There is general call for more research, policy, and programming attention that meet the reproductive health needs of young adult African women [25,26,4]. Therefore, the aim of this multi-country study was to assess the influence of female adolescents’ reproductive health decision-making capacity on their contraceptive usage.

We anticipate that female adolescents’ reproductive health decision-making capacity would shape their sociocultural norms (e.g., subjective gender role orientations) and positively be related to contraceptive use.

# Materials and methods

## Data source

The study made use of pooled data from current Demographic and Health Survey (DHS) conducted from January 1, 2010 and December 31, 2018 in 32 countries in sub- Saharan Africa.

Table 1 provides a full list of the countries included in the survey and their survey years. The 32 countries were used in the study because they had complete data on the variables of interest for this study. The study included the countries under the DHS program in order to provide a holistic and in-depth evidence of the relationship between reproductive health decision making capacity and contraceptive use in SSA. DHS is a nationwide survey collected every five-year period across low and middle-income countries. The survey is representative of each of these countries. Women’s files were used for our study and these files possess the responses by women aged 15–49. The survey targets core maternal and child health indicators such as unintended pregnancy, contraceptive use, skilled birth attendance, immunisation among underfives, and intimate partner violence. Stratified dual-stage sampling approach was employed and the same questions were posed to all women making it feasible for multi-country study. Selection of clusters (i.e., enumeration areas [EAs]) is the first step in the sampling process followed by systematic household sampling within the selected EAs. For the purpose of this study, only sexually active adolescent girls (15-19 years) who had complete cases on all the variables of interest were used for the study (N = 11,474). Detailed description of the study sample is shown in Table 1.

### Table 1: Description of the study sample

| **Country** | **Survey Year** | **Sample (N)** | **Sample (%)** |
| --- | --- | --- | --- |
| Angola | 2015-16 | 660 | 19.6 |
| Burkina Faso | 2010 | 299 | 8.9 |
| Benin | 2017-18 | 273 | 8.2 |
| Burundi | 2016-17 | 204 | 5.1 |
| Congo DR. | 2013-14 | 541 | 13.6 |
| Congo | 2011-12 | 384 | 17.8 |
| Côte d'Ivoire | 2011-12 | 194 | 9.7 |
| Cameroon | 2011 | 170 | 4.7 |
| Ethiopia | 2016 | 661 | 18.9 |
| Gabon | 2012 | 233 | 12.7 |
| Ghana | 2014 | 66 | 3.4 |
| Gambia | 2013 | 17 | 0.7 |
| Guinea | 2018 | 677 | 26.4 |
| Kenya | 2014 | 215 | 3.5 |
| Comoros | 2012 | 142 | 11.0 |
| Liberia | 2013 | 278 | 14.5 |
| Lesotho | 2014 | 48 | 3.1 |
| Mali | 2018 | 888 | 40.2 |
| Malawi | 2015-16 | 1181 | 22.4 |
| Mozambique | 2015 | 346 | 22.3 |
| Nigeria | 2018 | 731 | 8.7 |
| Niger | 2012 | 316 | 16.6 |
| Namibia | 2013 | 92 | 5.0 |
| Rwanda | 2014-15 | 41 | 1.5 |
| Sierra Leone | 2013 | 411 | 10.1 |
| Senegal | 2010-11 | 369 | 10.2 |
| South Africa | 2016 | 38 | 2.5 |
| Chad | 2014-15 | 86 | 2.2 |
| Togo | 2013-14 | 127 | 7.3 |
| Uganda | 2016 | 867 | 20.3 |
| Zambia | 2013-14 | 550 | 14.9 |
| Zimbabwe | 2015 | 379 | 17.6 |
| Total |  | 11,474 | 100.0 |

## Study variables

### Dependent variable

The dependent variable employed for this study was “contraceptive use” which was derived from ‘current contraceptive method’. The responses were coded 0 = “No method”, 1 =

“folkloric method”, 2= “traditional method” and 3 = “modern method”. The existing DHS variable excluded women who were pregnant and those who had never had sex. The modern methods included female sterilization, male sterilization, intrauterine contraceptive device

(IUD), contraceptive injection, contraceptive implants (Norplant), contraceptive pill, condoms, emergency contraception, standard day method (SDM), vaginal methods (foam, jelly, suppository), lactational amenorrhea method (LAM), country-specific modern methods and respondent-mentioned other modern contraceptive methods (including cervical cap, contraceptive sponge, and others). Periodic abstinence (rhythm, calendar method), withdrawal (coitus interruptus) and country-specific traditional methods of proven effectiveness were considered as traditional methods whiles locally described methods and spiritual methods of unproven effectiveness, such as herbs, amulets, gris-gris, etc. were the folkloric methods. To obtain a binary outcome, all respondents who said they used ‘no method’ were put in one category and were given the code ‘0 = No’ whereas those who were using either folkloric, traditional or modern method were also put into one category and given the code ‘1= Yes’.

### Explanatory variables

The main explanatory variable was reproductive health decision-making capacity. Two derivative variables that focused on decision-making on sexual intercourse and condom use were done. For the decision-making on sexual intercourse, female adolescents were asked if they can refuse their partners sex while for the decision-making on condom use, female adolescents were asked if they can ask their partners to use condoms during sexual intercourse. Reproductive health decision-making capacity was then generated from the combination of the decision-making on sexual intercourse and condom use variables. Following previous studies on reproductive health decision-making capacity [18,19], the original response category of these variables (1 = yes, 2 = no and 3 = don’t know/ not sure) were re-categorised whereby “no and don’t know/not sure” were recoded as “0” with “yes” recoded as 1. Responses coded as

“0” were considered as not capable in making reproductive health decision whilst those coded

as “1" were labelled as capable of making reproductive health decisions.

Apart from the main explanatory variable, eight other variables were considered in our study as covariates. These variables were age, place of residence, wealth quintile, occupation, educational level, age at first sex, and survey country. Again, apart from survey country, these variables were not determined a priori, instead their selection was based on their significant association with the outcome variable–contraceptives use, as reported in previous studies [27,28]. Four of the covariates were recoded to make them meaningful for analysis and interpretation. Marital status was recoded into ‘cohabiting (1)’ and ‘married (2)’. Occupation was captured as ‘not working (0)’ and ‘working (1)’. Educational level was recoded as ‘no education (0)’, ‘primary (1)’ and ‘secondary/higher (2)’. Age at first sex was coded as ‘less than 16 years (1)’and ‘16-19 years (2)’.

## Statistical analysis

The analysis begun with computation of contraceptive prevalence and proportion of female adolescents who could ask refuse a partner sex, ask a partner to use condom during sex and those who have the capacity to make reproductive health decisions in the 32 SSA countries.

The syntax “metan” in STATA version 14.0 (StataCorp, College Station, TX, USA) was used to generate forest plots for each of these indicators. Each forest plot showed the prevalence of an indicator in individual countries and its corresponding weight, as well as the pooled prevalence in all the countries and its associated 95% confidence intervals (CI). A test of heterogeneity of the DHS data obtained for the different countries showed a high level of inconsistency (I^2^ > 50%) thereby warranting the use of a random effect model in all the metaanalysis (see Figures 1, 2, 3 and 4). Secondly, we appended the datasets and generated a total sample of 11,474. After appending, we calculated contraceptive prevalence across the sociodemographic characteristics with their significance levels and chi square values [χ^2^]. Multicollinearity test was performed and with a mean VIF of 1.21, there was no evidence of multicollinearity between the variables (see S1 Table). Using the explanatory variables which were significantly associated with contraceptive use (p<0.05) among female adolescents from the chi-square test, binary logistic regression analysis was then carried out in a hierarchical order where Model I looked at a bivariate analysis of the main independent variable

[reproductive health decision-making capacity] and the outcome variable [contraceptive use]. Model II, on the other hand, looked at a complete model of all the explanatory variables and the outcome variable [see Table 3]. In line with research evidence that modern contraceptives are the most effective contraceptives [29,30,31], a further analysis was done to examine the influence of reproductive health decision-making capacity on modern contraceptive use (see

Table 4). All frequency distributions were weighted while the survey [svy] command in STATA version 14.2 was used to adjust for the complex sampling structure of the data in the regression analyses. Results for the regression analysis have been presented as Crude Odds Ratios [COR] and Adjusted Odds Ratios (AOR) with their corresponding 95% confidence intervals [CI] signifying precision and significance of the reported OR values. Statistical significance was declared at p<0.05.

## Ethical Approval

The DHS surveys obtain ethical clearance from the Ethics Committee of ORC Macro Inc. as well as Ethics Boards of partner organisations of the various countries such the Ministries of Health. During each of the surveys, either written or verbal consent was provided by the women. Since the data was not collected by the authors of this manuscript, official permission was sought from MEASURE DHS website and access to the data was provided after our intent for the request was assessed and approved on 3rd April, 2019. Data is available on [https://dhsprogram.com/data/available-datasets.cfm.](https://dhsprogram.com/data/available-datasets.cfm)

# Results

## Percentage scores on the prevalence of contraceptive use within selected SSA countries

The prevalence of contraceptive use in each of the 32 SSA countries included in the study are presented in Figure 1. The overall prevalence of contraceptive use was 20.07% (95% CI: 16.1424.01), ranging from 2.30% (95% CI:0.87-5.47) in Chad to 48.8% (95% CI: 33.50-64.10) in Rwanda.

**Fig 1: Contraceptive prevalence among female adolescents in SSA**

### Proportion of female adolescents who can refuse sex to a partner

Figure 2 presents results on the proportion of female adolescents in SSA who could refuse sex to their partners. The results indicate that 62.25% (95% CI=55.75-68.76) of female adolescents could refuse sex to their partners in SSA. Majority of female adolescents who could refuse their partners sex were in Cameroon [62.25% (95% CI=55.75-68.76)] whiles the smallest proportion was in Senegal [27% (95% CI=22.47-31.53)].

### Fig 2: Proportion of female adolescents in SSA who can refuse their partners sex Proportion of female adolescents who can ask their partners to use condom during sex

Figure 3 presents results on the proportion of female adolescents in SSA who could ask their partners to use condom during sex. Overall, 56.95% (95% CI=48.47-65.42) of female adolescents could ask their partners to use condom during sex in SSA. Out of this number, majority of them were in Namibia [91.30% (95% CI=85-54-97.06)] whiles a few of them were in Niger [18% (95% CI=13.76-22.24)].

**Fig 3: Proportion of female adolescents in SSA who can ask their partners to use condom during sex**

### Proportion of female adolescents in SSA who have the capacity to make reproductive health decisions

Figure 4 presents results on the proportion of female adolescents in SSA who have the capacity to make reproductive health decisions. Overall, 71.97% (95% CI: 65.48-78.46) of female adolescents in all the 32 SSA countries had the capacity to make reproductive health decisions.

Female adolescents in Niger had the lowest reproductive health decision-making capacity [32.90% (95% CI: 27.72-38.08] whiles those in Lesotho had the highest proportion of reproductive health decision-making capacity [95.80% (95% CI: 90.13-101.47].

**Fig 4: Proportion of female adolescents in SSA who have the capacity to make reproductive health decisions**

## Chi-square results on contraceptive use across socio-demographic characteristics

Table 2 provides a summary the proportion of contraceptive use across the selected sociodemographic characteristics. Nineteen-year-old adolescents had a greater proportion of contraceptive use [25.3%]. Contraceptive use was high among female adolescents in urban areas [24.2%] compared to those in rural areas [17.9%]. With wealth quintile, female adolescents with the richest wealth quintile had the highest proportion of contraceptive use [28.1%]. In terms of occupation, female adolescents who were working had the highest contraceptive prevalence [20.1%]. Female adolescents who were cohabiting recorded the highest proportion of contraceptive use [22.6%] compared to those who were married [18.2%]. Female adolescents with secondary/higher education [28.0%] and those whose age at first sex ranged between 16 and 19 years [20.6%] had the highest proportion of contraceptive use with respect to education level and age at first sex respectively. Apart from occupation, all the explanatory variables showed statistically significant associations with contraceptive use among female adolescents at 95% confidence interval (see Table 2).

### Table 2: Socio-demographic characteristics and contraceptive use among female adolescents in SSA

| **Variables** | **Weighted N** | **Weighted %** | **Contraceptive use** | |
| --- | --- | --- | --- | --- |
| **Age [***χ^2^*=182.3, p<0.001] |  |  | **N** | **%** |
| 15 | 477 | 4.2 | 44 | 8.5 |
| 16 | 1,067 | 9.3 | 129 | 11.8 |
| 17 | 1,935 | 16.9 | 319 | 16.6 |
| 18 | 3,836 | 33.3 | 706 | 18.6 |
| 19 | 4,169 | 36.3 | 1,050 | 25.3 |
| **Residence [***χ^2^*=56.0, p<0.001] |  |  |  |  |
| Urban | 3,011 | 26.2 | 733 | 24.2 |
| Rural | 8,463 | 73.8 | 1,515 | 17.9 |
| **Wealth Quintile [***χ^2^*=101.0, p<0.001] | |  |  |  |
| Poorest | 2,797 | 24.4 | 501 | 16.0 |
| Poorer | 2,856 | 24.9 | 505 | 17.7 |
| Middle | 2,528 | 22.0 | 463 | 19.5 |
| Richer | 2,023 | 17.6 | 433 | 22.9 |
| Richest | 1,270 | 11.1 | 346 | 28.1 |
| **Occupation [***χ^2^*=1.8, p>0.05] |  |  |  |  |
| Not working | 5,040 | 43.9 | 1,063 | 19.1 |
| Working | 6,434 | 56.1 | 1,185 | 20.1 |
| **Marital Status [***χ^2^*=30.3, p<0.001] | |  |  |  |
| Married | 7,977 | 69.5 | 1,429 | 18.2 |
| Cohabiting | 3,497 | 30.5 | 819 | 22.6 |
| **Educational Level [***χ^2^*=545.5, p<0.001] | |  |  |  |
| No Education | 3,389 | 29.5 | 230 | 6.7 |
| Primary | 4,906 | 42.8 | 1,167 | 23.4 |
| Secondary | 3,178 | 27.7 | 851 | 28.0 |
| **Age at First Sex [***χ^2^*=5.5, p<0.05] | |  |  |  |
| Less than 16 years | 6,395 | 55.7 | 1,233 | 18.8 |
| 16-19 years | 5,079 | 44.3 | 1,015 | 20.6 |

Source: Authors’ computations

## Binary Logistic regression analysis on female adolescents’ health decision-making capacity and contraceptive use

Results in Table 3 on reproductive health decision-making capacity and contraceptive use showed that female adolescents who had the capacity to make reproductive health decisions were more likely to use contraceptives [OR = 2.41; CI = 2.15 – 2.71, p < 0.001] compared to those who did not have the capacity to make reproductive health decision, even after controlling for the effect of the covariates [AOR = 1.41; CI = 1.24–1.60, p < 0.001]. Compared to Chad, female adolescents in Zimbabwe had the highest odds of using contraceptives [AOR = 36.50; CI = 8.98–148.3, p < 0.001]. The odds of contraceptive use among female adolescents increased with age, with those aged 19 years having the highest likelihood of using contraceptives [AOR = 3.59; CI = 2.56–5.04, p < 0.001]. The results further showed that the higher the level of education, the more likely some female adolescents will use contraceptives and this was more predominant among individuals with secondary education [AOR = 2.46; CI = 2.04–2.98, p < 0.001].

Female adolescents who were cohabiting had higher odds of using contraceptives compared to those who were married [AOR = 1.57; CI = 1.34–1.83, p < 0.001]. The odds of contraceptive use was highest among female adolescents with richest wealth quintile compared to those with poorest wealth quintile [AOR = 1.51; CI = 1.22–1.87, p < 0.001]. Female adolescents in rural areas were less likely to use contraceptives compared to those in urban areas [AOR = 0.77; CI = 0.67–0.89, p < 0.001]. Regarding age at first sex, female adolescents whose age at first sex was between 16 and 19 years were less likely to use contraceptives compared to those whose age at first sex was less than 16 years [[AOR = 0.64; CI = 0.58–0.72], p < 0.001](see Table 3).

### Table 3: Multivariable analysis on determinants of contraceptive use among female adolescents in SSA

| **Variables** | **Model I**  **COR[95%CI]** | **Model II**  **AOR[95%CI]** |
| --- | --- | --- |
| **Reproductive health decisionmaking capacity** |  |  |
| Incapable | Ref | Ref |
| Capable | 2.41^***^[2.15–2.71] | 1.41^***^[1.24–1.60] |
| **Country** |  |  |
| Angola |  | 1.92 [0.46–7.99] |
| Burkina Faso |  | 7.09^**^ [1.70–29.5] |
| Benin |  | 3.46 [0.80–14.99] |
| Burundi |  | 11.13^***^ [2.68–46.26] |
| Congo Dr. |  | 3.98 [0.97–16.33] |
| Congo |  | 15.60^***^ [3.81–63.87] |
| Côte d'Ivoire |  | 7.02^**^ [1.65–29.80] |
| Cameroon |  | 7.90^**^ [1.88–33.14] |
| Ethiopia |  | 18.58^***^ [4.62–74.76] |
| Gabon |  | 8.99^**^ [2.16–37.43] |
| Ghana |  | 7.98^**^ [1.78–35.87] |
| Gambia |  | 5.15 [0.72–36.63] |
| Guinea |  | 5.88^*^ [1.44–24.03] |
| Kenya |  | 17.52^***^[4.26–72.18] |
| Comoros |  | 10.69^**^[2.53–45.25] |
| Liberia |  | 2.94 [0.69–12.46] |

| Lesotho |  | 17.21^***^[3.83–77.33] |
| --- | --- | --- |
| Mali |  | 6.66^**^ [1.64–26.98] |
| Malawi |  | 30.52^***^[7.62–122.2] |
| Mozambique |  | 6.16^*^ [1.50–25.34] |
| Nigeria |  | 2.95 [0.71–12.25] |
| Niger |  | 8.26^**^ [1.97–34.62] |
| Namibia |  | 16.46^***^ [3.80–71.29] |
| Rwanda |  | 22.72^***^ [4.92–105.0] |
| Sierra Leone |  | 3.96 [0.96–16.42] |
| Senegal |  | 4.51^*^ [1.05–19.28] |
| Chad |  | Ref |
| Togo |  | 3.93 [0.87–17.64] |
| Uganda |  | 8.21^**^ [2.03–33.23] |
| South Africa |  | 28.29^***^ [6.02–132.9] |
| Zambia |  | 22.12^***^ [5.48–89.27] |
| Zimbabwe |  | 36.50^***^[8.98–148.3] |
| **Age** |  |  |
| 15 |  | Ref |
| 16 |  | 1.44[0.99–2.09] |
| 17 |  | 2.27^***^[1.59–3.22] |
| 18 |  | 2.80^**^[1.99–3.93] |
| 19 |  | 3.59^***^[2.56–5.04] |
| **Place of residence** |  |  |
| Urban |  | Ref |
| Rural |  | 0.77^***^[0.67–0.89] |
| **Educational level** |  |  |
| No Education |  | Ref |
| Primary |  | 1.97^***^[1.66–2.34] |
| Secondary/Higher |  | 2.46^***^[2.04–2.98] |
| **Marital status** |  |  |
| Married |  | Ref |
| Cohabiting |  | 1.57^***^[1.34–1.83] |
| **Wealth quintile** |  |  |
| Poorest |  | Ref |
| Poorer |  | 1.12[0.96–1.29] |
| Middle |  | 1.21^*^[1.04 –1.41] |
| Richer |  | 1.34^**^[1.13–1.60] |
| Richest |  | 1.51^***^[1.22–1.87] |
| **Age at First Sex** |  |  |
| Less than 16 years |  | Ref |
| 16-19 years |  | 0.64^***^[0.58–0.72] |
| *N* | 11,474 | 11,474 |
| pseudo *R*^2^ | 0.023 | 0.155 |

314

315 * p<0.05, ** p<0.01, *** p<0.001; Ref=Reference , CI=Confidence Intervals , COR=Crude
316 Odds Ratio, AOR=Adjusted Odds Ratio
317 Source: Authors’ computations

## Binary Logistic regression analysis on female adolescents’ health decision-making capacity and modern contraceptive use

Results in Table 4 on reproductive health decision-making capacity and modern contraceptive use showed that female adolescents who had the capacity to make reproductive health decisions were more likely to use modern contraceptives [OR = 2.29; CI = 2.03 – 2.58], p < 0.001] compared to those who did not have the capacity to make reproductive health decision, even after controlling for the effect of the covariates [AOR = 1.38; CI = 1.21–1.58], p < 0.001]. Compared to Chad, the highest odds of modern contraceptives use was among female adolescents in Zimbabwe [AOR = 34.97; CI = 8.61–142.1, p < 0.001]. The likelihood of modern contraceptive use among female adolescents increased with age, with those aged 19 years having the highest likelihood of using contraceptives [AOR = 3.24; CI = 2.26–4.63, p < 0.001]. The odds of modern contraceptives usage were low among female adolescents in rural areas compared to those in urban areas [AOR = 0.72; CI = 0.63–0.84, p < 0.001]. The results further showed that the higher the level of education, the more likely some female adolescents will use modern contraceptives and this was more predominant among individuals with secondary education [AOR = 2.49; CI = 2.03–3.05, p < 0.001].

Female adolescents who were cohabiting were more likely to use modern

contraceptives compared to those who were married [AOR = 1.48; CI = 1.26–1.75, p < 0.001]. The odds of modern contraceptive use was highest among female adolescents with richest wealth quintile compared to those with poorest wealth quintile [AOR = 1.45; CI = 1.16–1.81], p < 0.001]. Regarding age at first sex, female adolescents whose age at first sex was between 16 and 19 years were less likely to use modern contraceptives compared to those whose age at first sex was less than 16 years [AOR = 0.63; CI = 0.58–0.71, p < 0.001](see Table 4).

342	**Table 4: Multivariable analysis on determinants of modern contraceptive use among**

### 343 female adolescents in SSA

| **Variables** | **Model I**  **COR[95%CI]** | **Model II**  **AOR[95%CI]** |
| --- | --- | --- |
| **Reproductive health decisionmaking capacity** |  |  |
| Incapable | Ref | Ref |
| Capable | 2.29^***^ [2.03–2.58] | 1.38^***^ [1.21–1.58] |
| **Country** |  |  |
| Angola |  | 1.76 [0.42–7.36] |
| Burkina Faso |  | 6.403^*^[1.53–26.74] |
| Benin |  | 2.82 [0.64–12.37] |
| Burundi |  | 9.42^**^ [2.26–39.37] |
| Congo Dr. |  | 1.27 [0.30–5.43] |
| Congo |  | 7.19^**^[1.74–29.64] |
| Côte d'Ivoire |  | 3.17 [0.71–14.16] |
| Cameroon |  | 4.98^*^[1.17–21.18] |
| Ethiopia |  | 17.98^***^[4.47–72.32] |
| Gabon |  | 5.48^*^ [1.30–23.04] |
| Ghana |  | 6.70^*^ [1.47–30.54] |
| Gambia |  | 4.94 [0.69–35.29] |
| Guinea |  | 5.53^*^ [1.35–22.61] |
| Kenya |  | 16.04^***^[3.89–66.12] |
| Comoros |  | 6.61^*^ [1.53–28.50] |
| Liberia |  | 3.00 [0.71–12.73] |
| Lesotho |  | 17.10^***^ [3.81–76.80] |
| Mali |  | 5.89^*^[1.45–23.88] |
| Malawi |  | 29.75^***^[7.43–119.2] |
| Mozambique |  | 5.91^*^ [1.43–24.32] |
| Nigeria |  | 1.96 [0.47–8.29] |
| Niger |  | 7.47^**^ [1.78–31.37] |
| Namibia |  | 15.14^***^[3.49–65.74] |
| Rwanda |  | 23.98^***^[5.18–110.9] |
| Sierra Leone |  | 3.94[0.95–16.34] |
| Senegal |  | 3.44 [0.79–14.92] |
| Chad |  | Ref |
| Togo |  | 3.29 [0.72–15.07] |
| Uganda |  | 7.75^**^[1.91–31.39] |
| South Africa |  | 28.09^***^[5.97–132.1] |
| Zambia |  | 19.70^***^[4.88–79.51] |
| Zimbabwe |  | 34.97^***^[8.61–142.1] |
| **Age** |  |  |
| 15 |  | Ref |
| 16 |  | 1.37 [0.92–2.04] |
| 17 |  | 2.10^***^[1.44–3.04] |
| 18 |  | 2.51^***^[1.75–3.60] |

| 19 |  | 3.24^***^[2.26–4.63] |
| --- | --- | --- |
| **Place of residence** |  |  |
| Urban |  | Ref |
| Rural |  | 0.72^***^[0.63–0.84] |
| **Educational level** |  |  |
| No Education |  | Ref |
| Primary |  | 1.99^***^[1.65–2.39] |
| Secondary/Higher |  | 2.49^***^[2.03–3.05] |
| **Marital status** |  |  |
| Married |  | Ref |
| Cohabiting |  | 1.48^***^[1.26–1.75] |
| **Wealth quintile** |  |  |
| Poorest |  | Ref |
| Poorer |  | 1.10 [0.94–1.29] |
| Middle |  | 1.21^*^[1.03–1.42] |
| Richer |  | 1.30^**^[1.08–1.56] |
| Richest |  | 1.45^***^[1.16–1.81] |
| **Age at First Sex** |  |  |
| Less than 16 years |  | Ref |
| 16-19 years |  | 0.63^***^[0.58–0.71] |
| *N* | 11,474 | 11,474 |
| pseudo *R*^2^ | 0.02 | 0.164 |

* p<0.05, ** p<0.01, *** p<0.001; Ref=Reference , CI=Confidence Intervals , COR=Crude

Odds Ratio, AOR=Adjusted Odds Ratio

Source: Authors’ computations

# Discussions

This multi-country study assessed the influence of female adolescents’ reproductive health decision-making capacity on their contraceptive usage in 32 SSA. The study was critical since female’s ability to take decisive reproductive health decisions including choices in contraceptive use (i.e., condom among others) can lead to good reproductive health [19] and also the prevention of certain sexually transmittable infections (e.g. HIV, STI’s and Hepatitis among others) [26]. Generally, 71.97% of females across the 32 countries included in the study had the capacity (i.e., empowerment) to make reproductive health decisions, a finding that is similar but higher compared to 69.3% capacity found in other studies (e.g., [32]). This variation might be due to differences in the study populations and the number of countries these studies were based on. We found that females who had capacity to make reproductive health decisions were more likely to use contraceptives. This does not necessarily imply that females who had no capacity (i.e. empowerment) had no priority of contraceptive use. Some scholars have found that females’ restrictive roles in domestic matters including reproductive health issues in many sub-Saharan countries (e.g., Ghana, Kenya, Tanzania, Nigeria, Uganda and South Africa) rest with men [33], a reason that could derail them from contraceptive usage. Female adolescents from Zimbabwe had the highest odds of contraceptive usage compared to Angola with the least. This results is not surprising because Zimbabwe, together with other southern African states (e.g., Namibia) have one of the most effective family planning programs in sub-Saharan Africa (SSA) with lowest unmet need for contraception among adolescent women in Africa [34]. Other plausible reasons might be Zimbabwe’s positive success in female adolescents’ contraceptive usage and their high literacy rate imply that their adolescents might have capacity in many other areas of life such as socio-cultural and familial empowerment [35] compared to other young females in the other 31 SSA countries. What may also be accounting for these sub-Saharan societies with lower odds of using contraceptives could be ascribed to the low acceptance and high cultural resistance to family planning. According to [36], the social, financial, and strict kingship norms and values devoted to children and family in the region are also contributory toward the application of contraceptives.

An age progression in contraceptive usage, with those aged 19 years having the highest likelihood of using contraceptives in the current study. This finding corroborate previous studies in developing countries like Pakistan [37], Nepal [38] and Ghana [19] where females’ reproductive health decision making capacity increase with age as their other dimensions of empowerment increases. Female adolescents in rural dwellings were less likely to use contraceptives compared to their urban counterparts. This disparity in rural-urban contraceptive use could be due to limited and lack of access to health care services in rural communities, and even where they exist, socio-cultural limitations may hinder usage [39]. This assertion reinforces the rural-urban inequities in terms of essential services like reproductive health (i.e., family planning services) for female adolescents. This finding can best be appreciated when we reflect on how health facilities, key human and other resources are skewed in favour of urban settings in most countries across SSA [40]. African governments and non-governmental organisations could roll out reproductive health services, the adoption, and replication of Community Health Programme Services (CHPS) concept across the subregion to grant quality healthcare access to rural dwellers.

The higher the level of education, the more likely some female adolescents will use contraceptives, predominantly among those with secondary/higher education. Specifically, the odds of contraceptive use were higher for those with secondary and/ or higher education, compared with those with none, a finding similar to previous studies in Ghana and other low and middle-income countries [41,18]. This finding strongly implies that educating girls may be an effective tool for promoting reproductive health decision making capacity and contraceptive use. Educated females adolescents may have better access to health care information and reproductive health literature, better ability to use quality healthcare services and greater autonomy to make decisions [42]. Other studies have also proven that education may impart feelings of self-worth and self-confidence, which are necessary for changing health behaviour and seeking out health services for the first time [43].

The odds of contraceptive use were highest among female adolescents with richest wealth quintile compared to those with poorest wealth quintile. Consistent with previous studies in Africa [27] and elsewhere [44] that found women with high wealth index to have high affinity for contraceptive use and use of contraceptives. For example, [45] explained that

Ghanaian women who have high wealth status are more capable to take charge of their sexual and reproductive health matters (i.e. contraceptive use) than women of poorer wealth status. It is sound to argue that rich women who are but few in Africa, gain more repute, boost their confidence and are self-reliant to independently decide and afford any contraceptive of their choice. However, it is fair to extrapolate that females’ adolescents from the poorest wealth quintile may need to be more sexually empowered than their richer counterparts to overcome the greatest barriers accounting for the utilization of health services, including contraceptives.

African governments and gender empowerment NGO’s within the sub-region should target much of their social interventions (i.e. pro-poor) programs toward adolescent females, as most women in Africa are below the poverty line (evidence). Specific competency-based and life, skills-based education training interventions should be implemented across the sub-region by governments to broaden the scope of female adolescents’ reproductive health issues [46].

## Practical implications

Female adolescents’ ability to make reproductive health decisions was associated with contraceptive use. Nonetheless, it is imperative to recognize that female adolescents’ involvement in positive reproductive decisions is contingent strongly on the shared or interactive influence of their age, rural-urban geographical location, educational status, and socio-economic status. For most parts of SSA, many decisions on managing sexual and reproductive health have come under serious scrutinized from a socio-cultural lens as female adolescents’ vulnerability to unintended pregnancy, unsafe abortions, HIV and other STI’s have increased. For example, a deep rooted cultural barrier to contraceptive use in many parts of SSA could be the unfriendliness that many female adolescents experience when they visit maternal and child health clinics and other primary sources of contraceptives. Due to overt social dissatisfaction of premarital sexual activity and the general lack of confidentiality, and anonymity at these health centres, many young girls, by attempting to procure contraceptives, are exposed to public ridicule, gossip and negative attitudes from health personnel [47]. Therefore, female adolescents with lower reproductive health decision making capacity could be at high risk of mistreatment from health care providers, either by getting poor-quality services, or suspending health services all together [43].

Education, particularly secondary and/ or higher education, has repetitively been shown to be connected with a wide range of positive sexual reproductive health outcomes such as contraceptive use, age of marriage, number of births, and use of health services, albeit other proxies (e.g., socio-economic status). One systematic review of risk and protective factors for ASRH in low- and middle-income countries found that the more current in-school adolescents spend in school, the less likely they would ever engage in sex and have greater chances of using modern contraceptives compared to those who leave school early [48,49]. Other studies have proven that education may impart feelings of self-worth and self-confidence, which are necessary for changing health behaviour and seeking out health services for the first time [47]. Therefore, educational intervention programs that would promote the enrolment and retention of female adolescents in secondary schools and perhaps beyond are critical toward improving female adolescents’ reproductive health decision making capacity and their overall health.

Undoubtedly, though the DHS includes standard questions that allow multi-country comparisons, further in-depth qualitative enquiry and statistical linkages between different components of reproductive health decision making and adolescents’ likelihood to engage in sexual behaviours (e.g., onset of sexual debut, unsafe abortions, unintended pregnancies) would be helpful to better understand the context specific issues across sub-Sahara Africa. Critical consideration must be given to context specific institutional barriers (e.g., cultural norms and expectations for sex roles) to adolescents’ sexuality such as pregnancy prevention, healthcare providers' attitudes, and lack of confidentiality, and their impact on adolescents' capacity to make decisions about sexual and reproductive health issues [17].

## Strengths and Limitations

The main strength of the study is the use of national representative surveys whose methodology has been validated across the countries and as a result make the findings of our study valid and generalisable to other adolescents in SSA. Despite these strengths, the current study is not without limitations. First, the DHS data presented in this study is limited to 32 SSA countries and therefore may not be applicable to other countries and geographical settings. Second, this study is linked to the cross-sectional nature of DHS data. Given that the data for women's empowerment and contraceptive use were collected within a specific time frame, causal inferences or temporal relationship between studied variables cannot be ascertained. There is a probability that the similar observed or unobserved factors may impact both female adolescents’ reproductive health decision making capacity and contraceptive method used. For the purpose of robustness, some factors that are theoretically associated to contraceptive use but not to empowerment were statistically controlled, hence full testing and adjusting for endogeneity is necessary, but was beyond the scope of this study. Respondent needed to have answered ‘yes’ to both “ ability to refuse partners sex and ability to ask partner to use condom in order to be considered to have ‘reproductive health decision-making capacity. With this women using another contraceptive method might find it strange or difficult to request that their partner, particularly in longer-term or more serious relationships, use a condom (double protection). Second, the outcome variable, of contraceptive use is limited insufficiently nuanced to consider whether the adolescent has a need to avoid pregnancy. Also, the current study used the most recent DHS data, hence, variations in contraceptive accessibility and usage may have happened in the last few years. For example, countries like Zimbabwe and Namibia, have been promoting long-standing family planning services in some of their districts, whereas Zambia has seen a drastic shift in funding from family planning to HIV prevention and management. While these alterations are not likely to have caused changes in contraceptive use within a specific time frame, possible long term changes in these policy shifts may lead to increased usage of specific contraceptive methods (e.g., condom use) at the expense of others (i.e., IUD). Therefore, there may also be variations with the linkages between reproductive health decision making capacity and contraceptive use identified in this [42]. Lastly, due to the very sensitive nature of the sexuality themes under consideration, there is the possibility of social desirability concerns or recall bias among the study participants.

# Conclusions

Utilisation of contraceptives remains low in SSA among adolescents. This study revealed that reproductive health decision making capacity, age, education, wealth status, marital status, place of residence and age at first sex are associated with contraceptives use. It is therefore imperative to strengthen existing efforts in SSA on contraceptives use among adolescent by empowering adolescents to take reproductive health decisions, targeting younger adolescents, those in rural areas, those without formal education and those who initiate sexual intercourse between aged 16 and 19 years. These can help reduce teenage pregnancy and early childbirth, reduce maternal mortality and help in the achievement of SDG three. The current findings suggest that specific programs that aim to promote female adolescent's capacity to discuss sexual activity would be particularly be helpful in SSA countries (e.g., Chad, Gambia) that were identified with the lower odds of using contraceptives and where family planning services are limited. These programs should complement efforts to increase contraceptives’ availability to meet the rising demand for modern methods due to serious child and maternal health issues.

## Declaration of Conflicting Interests

The authors declared no potential conflicts of interest with the research, authorship, and/or publication of this article.

## Funding

The authors received no financial support for the research, authorship, and/or publication of this article.

# References

1. Blackstone SR, Nwaozuru U, Iwelunmor J. Factors influencing contraceptive use in subSaharan Africa: a systematic review. International quarterly of community health education. 2017 Jan; 37(2):79-91.
2. Doyle AM, Mavedzenge SN, Plummer ML, Ross DA. The sexual behaviour of adolescents in sub Saharan Africa: patterns and trends from national surveys. Tropical Medicine & International Health. 2012 Jul;17(7):796-807.
3. Chandra-Mouli, Venkatraman, Donna R. McCarraher, Sharon J. Phillips, Nancy E.

Williamson, and Gwyn Hainsworth. “Contraception for adolescents in low and middle income countries: Needs, barriers, and access,” Reproductive Health. 2014:11(1): 1–8. [https://doi.org/10.1186/1742-4755-11-1.](https://doi.org/10.1186/1742-4755-11-1)

4. Radovich, Emma, Mardieh L. Dennis, Kerry L.M. Wong, Moazzam Ali, Caroline A. Lynch, John Cleland, Onikepe Owolabi, Mark Lyons-Amos, and Lenka Benova. “Who meets the contraceptive needs of young women in Sub-Saharan Africa?” Journal of

Adolescent	Health.	2018:62(3):273–280.

[https://doi.org/10.1016/j.jadohealth.2017.09.013.](https://doi.org/10.1016/j.jadohealth.2017.09.013)

5. Tsui AO, Brown W, Li Q. Contraceptive practice in sub-Saharan Africa. Population and development review. 2017 May; 43(Suppl Suppl 1):166.
6. Bundy DA, de Silva N, Horton S, Patton GC, Schultz L, Jamison DT, ... & Appleby L. Investment in child and adolescent health and development: key messages from Disease Control Priorities. *The Lancet*. 2018:*391*(10121), 687-699.

7. MacQuarrie, Kerry L.D. “Unmet Need for Family Planning Among Young Women:

Levels and Trends [CR34].” Rockville, Maryland. 2014.

8. Presser HB. Demography, feminism, and the science-policy nexus. *Population and Development Review*. 1997:295-331.
9. Mason KO & Smith HL. Women’s empowerment and social context: Results from five Asian countries. *Gender and Development Group, World Bank, Washington, DC*. 2003
10. Kim JC, Watts CH. Gaining a foothold: tackling poverty, gender inequality, and HIV in Africa. Bmj. 2005 Sep 29;331(7519):769-72.
11. Blanc AK & Wolff B. Gender and decision-making over condom use in two districts in Uganda. *African Journal of Reproductive Health*. 2001:15-28.
12. Achan SF, Akweongo P, Debpuur C, Cleland J. Coping strategies of young mothers at risk of HIV/AIDS in the Kassena-Nankana district of Northern Ghana. African Journal of Reproductive Health. 2009;13(1).
13. Barnett B, Konaté M, Mhloyi M, Mutambirwa J, Francis-Chizororo M, Taruberekera N, Ulin P. The impact of family planning on women's lives: Findings from the Women's

Studies Project in Mali and Zimbabwe. African Journal of Reproductive Health.	1999;3(1):27-

14. Okal J, Stadler J, Ombidi W, Jao I, Luchters S, Temmerman M, Chersich MF. Secrecy, disclosure and accidental discovery: perspectives of diaphragm users in Mombasa,

Kenya. Culture, health & sexuality. 2008 Jan 1;10(1):13-26.

15. Blanc AK. The effect of power in sexual relationships on sexual and reproductive

health: an examination of the evidence. Studies in family planning. 2001	Sep;32(3):189-213.

16. Bankole A, Singh S. Couples' fertility and contraceptive decision-making in developing 570 countries: hearing the man's voice. International family planning perspectives. 1998 Mar 1:15-24.
17. Do M, Kurimoto N. Women's empowerment and choice of contraceptive methods in selected African countries. International perspectives on sexual and reproductive health. 2012 Mar 1:23-33.
18. Darteh EK, Doku DT, Esia-Donkoh K. Reproductive health decision-making among

Ghanaian women. Reproductive health. 2014;11(1):23. DC: FOCUS on Young Adults;

May 22.

19. Seidu AA, Ahinkorah BO, Agbemavi W, Amu H, Bonsu F. Reproductive health decision making capacity and pregnancy termination among Ghanaian women: Analysis of the 2014 Ghana demographic and health survey. Journal of Public Health. 2019:1-0.
20. Kulczycki A: The Abortion Debate in the World Arena. London: Macmillan Press Limited;

1999.

21. Oronje RN, Crichton J, Theobald S, Lithur NO, Ibisomi L, Oronje RN, Crichton J, Theobald S, Lithur NO, Ibisomi L: Operationalising sexual and reproductive and rights in sub-Saharan Africa: constraints, dilemmas and strategies. BMC Int Health Hum Rights 2011, 11(Suppl 3):S8.
22. UNFPA: ICPD 1994: Summary of the programme of action. Adv Physiol Educ 1994,

:1995.

23. Bankole A, Ahmed FH, Neema S, Ouedraogo C, Konyani S. Knowledge of correct condom use and consistency of use among adolescents in four countries in Sub-Saharan Africa.

African journal of reproductive health. 2007; 11(3):197.

24. Manlove J, Ikramullah E, Terry-Humen E. Condom use and consistency among male adolescents in the United States. Journal of adolescent health. 2008 Oct 1;43(4):325-

33.

25. Bearinger, Linda H., Renee E. Sieving, Jane Ferguson, and Vinit Sharma. “Global perspectives on the sexual and reproductive health of adolescents: Patterns, prevention, and potential,” The Lancet. 2007:369(9568): 1220–1231. https://doi.org/

10.1016/S0140-6736(07)60367-5.

26. Bankole A & Malarcher S. Removing barriers to adolescents' access to contraceptive information and services. *Studies in family planning*. 2010:*1*(2), 117-124.
27. Achana FS, Bawah AA, Jackson EF, Welaga P, Awine T, Asuo-Mante E, Oduro A, Awoonor-Williams JK, Phillips JF. Spatial and socio-demographic determinants of contraceptive use in the Upper East region of Ghana. Reproductive health. 2015 Dec;12(1):29.
28. Mandiwa C, Namondwe B, Makwinja A, Zamawe C. Factors associated with contraceptive use among young women in Malawi: analysis of the 2015–16 Malawi demographic and health survey data. Contraception and Reproductive Medicine. 2018 Dec;3(1):12.
29. Abiye, A. A., Fekede, B., Jemberie, A. M., Molla, B. A., Tolla, B. K., Tefera, B. S., ... & Kemal, H. S. (2019). Modern Contraceptive Use and Associated Factors among Reproductive Age Group Women in three Peri-Urban Communities in Central Ethiopia. *Journal of Drug Delivery and Therapeutics*, *9*(6-s), 93-102.
30. Ajayi, A. I., Adeniyi, O. V., & Akpan, W. (2018). Use of traditional and modern contraceptives among childbearing women: findings from a mixed methods study in two southwestern Nigerian states. *BMC public health*, *18*(1), 604.
31. Debebe, S., Limenih, M. A., & Biadgo, B. Modern contraceptive methods utilization and associated factors among reproductive aged women in rural Dembia District, northwest Ethiopia: Community based cross-sectional study. *International Journal of Reproductive BioMedicine*. 2017:*15*(6), 367.
32. Darteh EK, Dickson KS, Doku DT. Women’s reproductive health decision-making: A multi-country analysis of demographic and health surveys in sub-Saharan Africa. PloS one. 2019;14(1).
33. Tolhurst R, Amekudzi YP, Nyonator FK, Squire SB, Theobald S. “He will ask why the child gets sick so often”: The gendered dynamics of intra-household bargaining over healthcare for children with fever in the Volta Region of Ghana. Social Science & Medicine. 2008 Mar 1;66(5):1106-17.
34. Ngome E, Odimegwu C. The social context of adolescent women’s use of modern contraceptives in Zimbabwe: a multilevel analysis. Reproductive health. 2014 Dec;11(1):64.
35. Na M, Jennings L, Talegawkar SA, Ahmed S. Association between women’s empowerment and infant and child feeding practices in sub-Saharan Africa: an analysis of Demographic and health surveys. Public Health nutrition. 2015 Dec;18(17):3155-65.
36. Caldwell JC, Caldwell P. The cultural context of high fertility in sub-Saharan Africa.

Population and development review. 1987 Sep 1:409-37.

37. Saleem S, Bobak M. Women's autonomy, education and contraception use in Pakistan: a national study. Reproductive health. 2005 Dec;2(1):8.
38. Acharya DR, Bell JS, Simkhada P, Van Teijlingen ER, Regmi PR. Women's autonomy in household decision-making: a demographic study in Nepal. Reproductive health. 2010

Dec;7(1):15.

39. Muanda MF, Ndongo GP, Messina LJ, Bertrand JT. Barriers to modern contraceptive use in rural areas in DRC. Culture, health & sexuality. 2017 Sep 2;19(9):1011-23.
40. Yaya S, Bishwajit G, Gunawardena N. Socioeconomic factors associated with choice of delivery place among mothers: a population-based cross-sectional study in Guinea- Bissau. BMJ global health. 2019 Apr 1;4(2):e001341.
41. Atiglo DY, Codjoe SN. Meeting women’s demand for contraceptives in Ghana: Does autonomy matter?. Women & health. 2019 Apr 21;59(4):347-63.
42. Apanga PA, Adam MA. Factors influencing the uptake of family planning services in the Talensi District, Ghana. Pan African Medical Journal. 2015;20(1).
43. Singh, K., Bloom, S, Brodish, P. Gender equality as a means to improve maternal Social Sci Med 2013; 63: 2242-54.
44. Aslam SK, Zaheer S, Qureshi MS, Aslam SN, Shafique K. Socio-economic disparities in use of family planning methods among Pakistani women: findings from Pakistan demographic and health surveys. PloS one. 2016 Apr 7;11(4):e0153313.
45. Nyarko SH. Unintended Pregnancy among Pregnant Women in Ghana: Prevalence and Predictors. Journal of Pregnancy. 2019.
46. Ahinkorah BO, Hagan Junior JE, Seidu AA, Mintah JK, Sambah F, Schack T, Hormenu T. Examining Pregnancy Related Socio-Cultural Factors Among Adolescent Girls in the

Komenda-Edina-Eguafo-Abrem Municipality in the Central Region of Ghana: A Case Control Study. Frontiers in public health. 2019;7:93.

47. Senderowitz J. Health facility programs on reproductive health for young adults. Washington, DC: FOCUS on Young Adults; 1997 May 22.
48. Grown C, Gupta GR, Pande R. Taking action to improve women’s health through gender equality and women’s empowerment. The Lancet. 2005. Feb5;365(9458):541-3.
49. Mmari K, Sabherwal S. A review of risk and protective factors for adolescent sexual and reproductive health in developing countries: an update. Journal of Adolescent Health.

2013 Nov 1;53(5):562-72

Response to Reviewers

**AUTHOR’S RESPONSE TO REVIEWS**

**Title: Female adolescents’ reproductive health decision-making capacity and contraceptive use in sub-Saharan Africa: What does the future hold?**

## Dear Editor and Reviewers,

Thank you for your email dated 9th March 2020 enclosing the reviewer’s comments. On behalf of all authors, I convey our gratitude to you for the critical and constructive review that has led to the massive improvement of our paper entitled *“***Female adolescents’ reproductive health decision-making capacity and contraceptive use in sub-Saharan Africa: What does the future hold?***”.* We have carefully reviewed the comments from all the three reviewers and have revised the manuscript accordingly. Our responses are given in a point-by-point manner below.Most of the changes have been indicated in yellow colour. We believe the manuscript has improved substantively and will be published in your reputable journal.

**Version 1: PONE-D-19-29601**

## Date:4/4/2020

**REVIEWER #1:**

**Comment:** In the methodology, the author/authors suggested the use of Bivariate and multivariable analysis which include Pearson chi-square tests and binary logistic regression respectively, however, binary logistic regression model in most cases is misleading in interpreting the complex relationships of this nature. The authors need to demonstrate how they deal with existence of multicollinearity problems which obviously made the model deficiency. I feel that the problem of multicollinearity cannot be undermined in this study because it can lead the authors into making incorrect conclusion about relationships between responsive and explanatory variables. Therefore, I would suggest that the authors should consider adopting Principle component analysis (PCA) in addition to regression approaches to avoid this problem. The PCA is a multivariate technique which help to understand the underlying data structure and to form a smaller number of uncorrelated new variables. In other words, PCA reduces number of predictors to avoid multicollinearity problem and it is highly recommended due to its ability to identify a small number of derived variables from a larger number of original variables in order to simplify the subsequent analysis of the data.

***Response: Before results were interpreted, multicollinearity between explanatory variables was assessed through the variance inflation factors (VIF) at a reference value of 5 (We have attached this as a supplementary file. (page 7)***

**REVIEWER #2:**

I acknowledge the authors for a comprehensive and in terms of language, a well-written paper. The paper was easy to follow. Common, yet important limitations of studies of such types involving sexual and reproductive health (SRH) were detailed in the “Limitation” section. Few comments I have are as follows:

**Comment 1.** Important things to mention in the last part of the “Introduction” section always are to briefly mention similar studies, what they lacked (the research gaps) and which gaps the current study aims to fulfil. This would make clear for the readers how the study is different from other studies with similar research objective. This important aspect is seen to be missing in the “Introduction” part. An example for it would be: In the material and method section, the authors mention to have followed previous studies (referenced 33 and 34) (Line no. 190). We see the topic of these two studies are similar to the current study, and study of such types are existent, however, the authors neither mention this study in the “Introduction” nor the gaps these studies had and the gaps that the current study attempts to cover. Another similar comment when the authors directly mention other two studies (reference 42, 43) (L.352) in the discussion and compare their results with them.

***Response: We have revised the introduction of our manuscript to take into consideration these issues (see Page 3-5).***

**Comment 2.** Although the study covered many aspects determining the reproductive decision making and contraceptive use, some interesting analysis could have been carried out additionally. I suggest a subgroup analysis grouping the countries with SRH or contraceptive intervention and the countries without. I believe that reproductive decision alone cannot facilitate contraceptive use (To explain, even if one has the reproductive decision making capacity but no availability of contraceptive, he/she would not be able to use contraceptives despite the will. On the other hand, if a person has very easy access to contraceptives, a slight decision making capacity would also be enough for contraceptive use).

***Response: Even though sexual and reproductive health interventions are not evenly distributed in sub-Saharan Africa, each country has instituted various policies to tackle contraceptive usage: Please see (***Liang, M., Simelane, S., Fillo, G. F., Chalasani, S., Weny, K., Canelos, P. S., ... & Michielsen, K. (2019). The state of adolescent sexual and reproductive health. *Journal of Adolescent Health*, *65*(6), S3-S15.

Melesse, D. Y., Mutua, M. K., Choudhury, A., Wado, Y. D., Faye, C. M., Neal, S., & Boerma, T.

(2020). Adolescent sexual and reproductive health in sub-Saharan Africa: who is left behind?. *BMJ Global Health*, *5*(1).; [https://www.afro.who.int/health-topics/sexual-and-reproductive-health)](https://www.afro.who.int/health-topics/sexual-and-reproductive-health)

**Comment:** 3. L: 258-270: I suggest mentioning the CI of the given results. (CI across different countries).

***Response: We have presented the results as forest plots and the results are now showing their respective 95% Cis (Please see Fig 1-4).***

***Minor comments:***

**Comment:** 1. I would suggest replacing the word “downward”(L. 119)

2. L.127-130, I would consider rewriting the sentence with minor changes to make it clearer (e.g. parental communication is not related to high risk of adolescent pregnancy but I believe poor parental communication is)

***Response: We have revised the background based on the recommendations made by Reviewer 3 and all these issues have been addressed (see Page 3-5).***

**Comment:** 3. L. 102: reference missing for –“ despite sizable body of literature”

***Response: We have revised the background based on the recommendations made by Reviewer 3 (see Page 3-5).***

**Comment:** 4. L.106, 358 etc. I would consider putting the reference at the end of the sentence or after some words or data from the study but not directly after one word – “According” or

“Similarly”. Eg: “According to a study, these persons ……. [18] “OR “According to a study

[18],	these	persons	…….	“

5. L. 332,334: I suggest restructuring the last part of the sentence.

***Response: We have taken note of this and revised some of these statements in the manuscript.***

**REVIEWER #3:**

The statistical analysis is fairly simple compared to the conceptual challenges presented in the study. There is one issue that may require the opinion of a statistician regarding the inclusion of country as a random effect in the model. Overall the study shines a lens on the important challenge of ensuring adolescents have the power to make decisions about their reproductive health, including their contraceptive use. However, the study is hampered by insufficient nuance in the consideration of both its main explanatory and outcome variables. The paper would be much strengthened by a more critical discussion and reflection on this. My main critiques, described in specific comments below, refer to refining the crude measure of reproductive health decision-making capacity, considering the contraceptive need (rather than just contraceptive use) of adolescents, defining the ‘sexually active’ adolescent population, and expanding the outcome to modern contraception (rather than just any contraception).

## Major issues Introduction

**Comment:** Overall, the Introduction is very long and could be written in more clear and accessible language. The authors should consider substantially shortening the Introduction and having a clear message for each paragraph. I suggest beginning with a very brief summary on trends/regional prevalence of adolescent contraceptive use prior to the discussion of women’s empowerment conceptualisation and measurement.

***Response: We have revised the background taking on board all the suggestions (see Page 3-***

***5).***

## Methods

**Comment:** How was the DHS for each country selected? Was only the most recent survey used? Please provide a full list of countries and survey years (also see note in Results).

**Response: We mentioned in the methods how each DHS was selected and have provided a Table that contains the countries and their survey years and the sample (See Table 1).**

**Comment:** Pg 7, line 168: “same questions were posed to all women making it feasible for multi-country 169 study.” This isn’t quite true. While there is a standard DHS questionnaire, some adaptations are made to each survey. Were the countries and surveys selected for inclusion in this analysis based on whether they had certain questions included?

**Response: The countries were selected because they had similar variables. “*The DHS contain both ‘core’ and optional questionnaires (modules). Core questionnaires cover basic demographic and health content, including marriage, fertility, family planning, reproductive health and child health, whereas additional modules contain special topics, including***

***maternal mortality, men’s survey, anthropometry (height and weight measurement), anaemia blood testing, gender/domestic violence, malaria, maternal mortality, tobacco use, chronic illnesses and other biomarkers” Consequently, each survey is tailored to the needs of a particular country while containing several basic components that are comparable across all countries ( Corsi, Neuman, Finlay & Subramanian, 2012 p.1606). Corsi, D. J., Neuman, M., Finlay, J. E., & Subramanian, S. V. (2012). Demographic and health surveys: a profile. International journal of epidemiology, 41(6), 1602-1613.***

**Comment:** Pg 8, line 172: How was the population of sexually active adolescent girls identified in the datasets? Are these women ‘in union’ or was this based on time since last sexual intercourse? How was the need for contraception considered in the analysis, as some sexually active adolescents may want to become pregnant?

## Response: The population for sexually active adolescents were determined by those who indicated they have ever had sex and mentioned their age at first sex as used in previous studies (Appiah et al 2020). Please we acknowledge the need for contraception as a limitation in our study. (see page 26)

**Comment:** Pg 8, line 178: What methods are included in modern methods? Did the authors consider examining the outcome of modern method of contraception, rather than just any method of contraception? Why was modern method of contraception not used as a (secondary) outcome, particularly as using a folkloric method could reflect a desire to avoid pregnancy but an inability to access a more effective method?

***Response: The modern methods included female sterilization, male sterilization, intrauterine contraceptive device (IUD), contraceptive injection, contraceptive implants (Norplant), contraceptive pill, condoms, emergency contraception, standard day method (SDM), vaginal methods (foam, jelly, suppository), lactational amenorrhea method (LAM), country-specific modern methods and respondent-mentioned other modern contraceptive methods (including cervical cap, contraceptive sponge, and others). Aside examining contraceptive use in general, we also examined the factors associated with modern contraceptive use (secondary outcome variable) (see Table 3 ).***

**Comment:** For the explanatory variable of ‘reproductive health decision-making capacity’, how were discordant answers between the two component variables handled? Did the respondent need to have answered ‘yes’ to both refuse partners for sex and condom use in order to be considered to have ‘reproductive health decision-making capacity’? It seems likely that women using another contraceptive method might find it strange or difficult to request that their partner, particularly in longer-term or more serious relationships, use a condom (double protection). Did the authors conduct further analysis of how each component of the binary capacity indicator performed separately in relation to the study outcome? The DHS also often asks questions on experience of violence and/or coercive sex. Did the authors consider other potential constructs to include in the indicator? Further discussion of the construction and limitations of this variable to capture the extremely complex concept of ‘reproductive health decision-making capacity’ is needed.

***Response: We followed the measurement of ‘reproductive health decision-making capacity’ by previous studies:***

***Darteh EK, Doku DT, Esia-Donkoh K. Reproductive health decision-making among Ghanaian women. Reproductive health. 2014;11(1):23***

***Darteh, E. K. M., Dickson, K. S., & Doku, D. T. (2019). Women’s reproductive health decision-making: A multi-country analysis of demographic and health surveys in subSaharan Africa. PloS one, 14(1)***

***Seidu AA, Ahinkorah BO, Agbemavi W, Amu H, Bonsu F. Reproductive health decisionmaking capacity and pregnancy termination among Ghanaian women: Analysis of the 2014 Ghana demographic and health survey. Journal of Public Health. 2019:1-0.***

***We have acknowledged limitation in the construction of ‘reproductive health decisionmaking capacity’ in our manuscript and recommend further studies to look at these limitations to refine its measurement (page 26.)***

**Comment:** Pg 9, line 212-221: When combining surveys in multi-country studies to produce an overall result for sub-Saharan Africa (or any region), it is often necessary to weight countryspecific estimates by the country’s population. This is so that the result from a very large country like Nigeria has a greater weight in the combined effect analysis than a very small country such as Comoros. (Previous multi-country studies explaining how to apply population weights:https://doi.org/10.1016/j.jadohealth.2017.09.013 or https://doi.org/10.1111/tmi.12597) Did the authors have a reason for not applying population weights, in addition the individual survey weights standard in all DHS analysis, in the calculation of regional statistics? Finally, I defer to a statistician, but wonder whether country is more appropriate as a random effect rather than a covariate in the models.

***Response: We applied both population weight and sample weights (see page 10) .Again a test of heterogeneity of the DHS data obtained for the different countries showed a high level of inconsistency (I^2^ > 50%) thereby warranting the use of a random effect model in all the meta-analysis (see Figures 1, 2, 3 and 4), but at the regression models as used in previous studies, country was considered a covariate:***

***Darteh, E. K. M., Dickson, K. S., & Doku, D. T. (2019). Women’s reproductive health decision-making: A multi-country analysis of demographic and health surveys in subSaharan Africa. PloS one, 14(1);***

***Ameyaw, E. K., Budu, E., Sambah, F., Baatiema, L., Appiah, F., Seidu, A. A., & Ahinkorah, B. O. (2019). Prevalence and determinants of unintended pregnancy in sub-Saharan Africa: A multi-country analysis of demographic and health surveys. PloS one, 14(8).***

## Results

**Comment:** Please provide a summary table of country, survey year, sample size of sexually active adolescents and proportion of sexually active adolescents out of all adolescents. Please also provide a summary table of the distribution of covariates. These tables can be either part of the main paper or in supplementary materials.

***Response: This has been provided. See Table 1.***

**Comment:** Figure 2: As the reproductive decision-making capacity is a simplistic measure, it would be helpful to provide estimates for each of the two components, either in the main paper or in supplementary materials.

***Response: We have provided the estimates in the forest plots. See Figure 2-4***

**Comment:** Pg 16, lines 299-300: This links to a question in the Methods section about the need for contraception. Married adolescents may well be desiring a pregnancy so have no need for contraception.

***Response: We have acknowledged this as a limitation in the paper (page 26.)***

## Discussion

**Comment:** Overall the Discussion section is far too long and would do well to more succinctly summarise the key findings and their implications. The limitations section is insufficient in examining the severe limitations of the indicators used for both explanatory and outcome variables. ‘Reproductive health decision-making capacity’ is a complex construct reduced to a binary variable based on two components. The outcome of contraceptive use is likewise insufficiently nuanced to consider whether the adolescent has a need to avoid pregnancy.

***Response: We have revised the discussion and acknowledged the limitations in measurement of both the explanatory and outcome variables( See page 21-27).***

**Comment:** Pg 21, lines 374-381: This is overly speculative and not backed up either by the results or this study or properly cited. Earlier sexual debut may itself be a marker of

‘empowerment’ or lack thereof, particularly if the first sex was coerced. The DHS has further questions that could have allowed a more nuanced of this construct. Second, as the population was sexually active adolescents age 15-19, many would only very recently have had their sexual debut. Why did the authors not use more nuanced categories for age of sexual debut, particularly since all the 15- year-olds in the analysis, by definition, would fall into the <16 years at sexual debut category. What is sexual debut as the binary variable here actually telling us?

**Response: We have revised the discussion.**

**Comment:** Pg 22, line 403-404: Here, and implied elsewhere in the paper, is the assertion that increasing education or empowering adolescent girls may be an effective tool for increasing adolescent contraceptive use, instead of a goal worthwhile in itself. For example: “It is imperative to strengthen existing efforts in SSA on contraceptives use among adolescent by empowering adolescents to take reproductive health decisions” (line 499-500). The authors would do well to engage more carefully in the discussion of empowerment as a means to an end (contraceptive use) rather than simply the end itself. In particular due to the paper’s outcome of contraceptive use, rather than met need for contraception, the implication is that all adolescents should be using contraception, rather than all adolescents having freedom and power to choose to use contraception if they wish. **Response: We have revised this section. See page 24)**

## Minor issues

**Comment:** Suggest a careful read as typos were noted.

**Response: We have reviewed and edited the manuscript extensively. The manuscript has also been reviewed by a native English speaker.**

## Introduction

**Comment:** Some stylistic edits needed, such as in page 4, line 94, to say ‘For example, Ann

Blanc [15] opined…’ rather than listing the numeric reference only. **Response: These issues have been rectified.**

## Methods

**Comment:** Discussion of data source and sampling could be shortened as readers can reference the individual surveys for details of the specific sampling procedures. **Response: This section has been shortened.**

**Comment:** Pg 9, line 197: Is education level based on highest level of completed education? How were these grouped into categories? As some younger adolescents may still be in education, how did the authors consider this in the analysis?

## Response: Thank you. This is on highest level of education completed

**Comment:** Pg 9, line 197: Is country of origin the same as the survey country? Or does this refer to DHS questions about migration?

**Response: This is referring to surveyed country. This has been clarified in the methods section of the manuscript.**

**Comment:** Pg 9, line 199: “based on their significant association with the outcome variable– contraceptives use” Was there a specific threshold used?

**Response: The threshold was that all those studies used nationally-representative survey data.**

**Comment:** Pg 9, line 203: How did the authors code the occupation status of respondents still in education (students)?

**Response: Education was captured based on highest level completed.**

## Results

**Comment:** Figure 1: How have the countries been sorted in some way for this bar chart? It might make more sense to sort alphabetically.

**Response: This has been presented with forest plot (see Figure 1-4).**

**Comment:** Table 1: This table could be simplified by showing a total n for each row (instead of a sample size for ‘no’ and for ‘yes’). Only the percentage of ‘yes’ is needed (as the ‘no’ for any contraceptive use is just the corresponding fraction). Please provide confidence intervals. **Response: This has been presented with forest plot (see Figure 1-4).**

**Comment:** Pg 16, line 290: Is there a reason the comparison or reference country is Angola? **Response: We have revised this. We have used the country with the lowest proportion (Zambia) as the reference category.**

## Discussion

**Comment:** Pg 19, line 323: Could the very different estimate be related to the crude measure of reproductive decision-making capacity measure used in this study? **Response: This section has been revised.**

**Comment:** Pg 23, line 430: “Female adolescents’ ability to make reproductive health decisions was associated with contraceptive use, which in turn could be connected with perceived unmet need.” This point about contraceptive use connected with perceived unmet need is confusing. **Response: This statement has been revised.**
